# External Stimuli on Neural Networks: Analytical and Numerical Approaches

**DOI:** 10.3390/e23081034

**Published:** 2021-08-11

**Authors:** Evaldo M. F. Curado, Nilo B. Melgar, Fernando D. Nobre

**Affiliations:** Centro Brasileiro de Pesquisas Físicas and National Institute of Science and Technology for Complex Systems, Rua Xavier Sigaud 150, Urca, Rio de Janeiro 22290-180, Brazil; evaldo@cbpf.br (E.M.F.C.); nilobarrantes@gmail.com (N.B.M.)

**Keywords:** neural networks, models of single neurons, artificial intelligence, nonlinear dynamical systems, 07.05.Mh, 87.85.dq, 87.19.ll, 87.18.Sn, 05.90.+m

## Abstract

Based on the behavior of living beings, which react mostly to external stimuli, we introduce a neural-network model that uses external patterns as a fundamental tool for the process of recognition. In this proposal, external stimuli appear as an additional field, and basins of attraction, representing memories, arise in accordance with this new field. This is in contrast to the more-common attractor neural networks, where memories are attractors inside well-defined basins of attraction. We show that this procedure considerably increases the storage capabilities of the neural network; this property is illustrated by the standard Hopfield model, which reveals that the recognition capacity of our model may be enlarged, typically, by a factor $10^{2}$. The primary challenge here consists in calibrating the influence of the external stimulus, in order to attenuate the noise generated by memories that are not correlated with the external pattern. The system is analyzed primarily through numerical simulations. However, since there is the possibility of performing analytical calculations for the Hopfield model, the agreement between these two approaches can be tested—matching results are indicated in some cases. We also show that the present proposal exhibits a crucial attribute of living beings, which concerns their ability to react promptly to changes in the external environment. Additionally, we illustrate that this new approach may significantly enlarge the recognition capacity of neural networks in various situations; with correlated and non-correlated memories, as well as diluted, symmetric, or asymmetric interactions (synapses). This demonstrates that it can be implemented easily on a wide diversity of models.

## 1. Introduction

Although the area of neural networks (NNs) has experienced impressive developments in the last few decades [1,2,3,4], essential characteristics and reactions of the brain are still far from being satisfactorily replicated in these models. This is an essential direction to pursue, since NNs were initially introduced in order to reproduce some of the primary functions of the brain [5], and their first main practical application was pattern recognition [6]. So far, NNs have been more successful in artificial intelligence applications, such as information and image processing, than in emulating the reactions of living beings in nature.

The remarkable works of Darwin and Wallace on evolutionary theory [7,8,9] were fiercely debated throughout the 19th century, but are now considered fundamental to our understanding the world. They are accepted by all scientists as a starting point for studying everything related to living beings, and all organs of living creatures were, of course, molded by evolution. In particular, the brain itself grew and developed under the action of natural selection; it works in accordance with its evolutionary history. But what is signified by the evolutionary history of the brain? It means the development, from generation to generation, from species to species, of a complex system with fast responses to external stimuli—a fundamental condition of survival. A living being needs to react, in many cases instantaneously, to escape from a predator, to catch prey, to avoid an accident, and many other situations. This implies that the nervous systems of animals were developed as fundamental tools, able to react promptly to changes in the environment.

After millions of years of the development of nervous systems, which generally increased in size and complexity, a concentrated region of nervous cells appeared—the primitive brain—which quickly enlarged its size; this was a breakthrough in the evolution of living beings. However, a further duty was yet to be fully developed. Living beings had to react quickly and more efficiently to changes in the environment—this task was at the origin of the brain and is recorded “au fer et au feu” in the way that it works. This stimuli-dependence of the brain is behind all of its assignments, the old ones (essentially instinctive reactions), as well as the biologically newer ones (e.g., those related to cognition). During the whole of its evolutionary history, pattern recognition was a basic attribute of the brain, allowing living beings to react appropriately. NN models should, therefore, incorporate some of the main features related to this; below we outline, briefly, the main procedure for pattern recognition in living beings.

(a) Living beings accumulate memories (past input patterns), that are stored in some way, e.g., using Hebb’s rule [10,11].
(b) Without external stimuli, they do not recognize any pattern, and remain in a noisy state.
(c) In the presence of an external stimulus that is associated with some stored pattern, they recognize that pattern; if the external stimulus has no relation to any stored pattern, nothing is recognized.
(d) If the external stimulus associated with some memory disappears, the effectivity of the recognized pattern decreases, becoming essentially null after some time delay. This induces a return to a noisy state.
(e) In line with a common feature in nature, whenever an external stimulus abruptly changes its pattern, the living being quickly adjusts to the new recognized pattern and away from the old one.
(f) These steps are followed repeatedly in the presence of each new stimulus, or sequences of stimuli.

Biologically realistic pattern-recognition NN models should reproduce the actions sketched above (at least, most of them), by following the route of new concepts in complex systems [12,13,14,15]. These systems essentially live at the chaos–order or ordered–disordered borderlines, and effective NN models should be defined at these borderlines; chaotic, ordered, or disordered regimes can never yield appropriate proposals for NNs.

Along these lines, we will introduce a stimuli-dependent neural-network model, and its effectiveness in pattern recognition will be demonstrated; certainly, similar scenarios can be constructed for other brain process. Stimuli will be defined in the form of an external local field, which has already shown its effectiveness in the suppression of chaos in a recurrent fully connected NN model [16]. Here, we will illustrate how this works using the standard Hopfield model. In the next section we briefly review the paradigmatic Hopfield model, define the class of attractor neural networks (ANNs), and analyze some of their limitations. In Section 3, we present our model, which incorporates several of the points sketched above; the procedure will be applied to the Hopfield model, for which analytical calculations are feasible. We also discuss the new concepts embodied in this approach, emphasizing its pattern storage capacity and showing this is substantially larger than the capacity of ANN models. Analytical calculations within a mean-field approximation, with special attention given to its zero-temperature (T=0) limit, are carried out in Section 4. In Section 5, we present results from numerical simulations of uncorrelated patterns at T=0, comparing some of these with the analytical results. The significant increase in pattern storage capacity, as compared to ANN models, is indicated by these numerical simulations. The ability of the model to react promptly to changes in the external stimuli—particularly in replicating actions (b)–(e) described above, which are crucial for living beings—is verified in Section 6. The good performance of the present proposal in the case of previously stored correlated patterns is reported in Section 7. In Section 8, we introduce site dilution into the model, revealing that it also functions well for asymmetric diluted synapses. In Section 9, we discuss our main results, emphasizing some of the advantages with respect to previous NN models, and propose potential applications; finally, in Section 10, we present our conclusions.

## 2. The Hopfield Model and Attractor Neural Networks

A breakthrough in the NN area occurred in the beginning of the 1980s, with the proposal of a model due to Hopfield [17] (known in the literature as the Hopfield model), which has attracted the attention of many during the last few decades [1,2,3]. Imitating ideas from random magnetic models, particularly those from the Ising spin-glass model [4,18,19], one makes the analogies, neurons↔spins and synapseintensities↔couplingconstants. In this way, the Hopfield model is defined by means of a Hamiltonian,

(1) $$H\left(t\right)=-\sum_{\left(i,j\right)}J_{ij}\sigma_{i}\left(t\right)\sigma_{j}\left(t\right),$$

with $\sigma_{i}\left(t\right)=\pm 1$ (i=1,2,…,N) representing the state of the *i*-th neuron at time *t* [5], activated or at rest. Moreover, $J_{ij}$ stands for the intensity of the synapses between neurons *i* and *j* (considered as symmetric), whereas $\sum_{\left(i,j\right)}$ denotes a sum over all distinct pairs of neurons, corresponding to a fully connected neural network. The intensity of the synapses $J_{ij}$ is expressed in terms of *p* stored memories $\left\{\xi_{i}^{\mu }\right\}$, according to Hebb’s rule [10,11],

(2) $$J_{ij}=\frac{1}{N}\sum_{\mu =1}^{p}\xi_{i}^{\mu }\xi_{j}^{\mu }\;\;\;\;\;\;\;\left(i\neq j\right).$$

These memories should remain fixed along the whole time evolution (i.e., are quenched variables) and are assumed to be orthogonal on average [for finite *N* there may occur overlaps between memories of $O(1/\sqrt{N})$]. The obey the equation

(3) $$P\left(\xi_{i}^{\mu }\right)=\frac{1}{2}\delta \left(\xi_{i}^{\mu }-1\right)+\frac{1}{2}\delta \left(\xi_{i}^{\mu }+1\right).$$

The definition of the Hamiltonian above has attracted the interest of many physicists, particularly because statistical-mechanics techniques could be applied [20,21,22,23]. However, an immediate question concerns the assumption of symmetric couplings $\left\{J_{ij}\right\}$, which is a serious obstacle to an appropriate emulation of the brain, since it is well-known that real synapses violate this symmetry, i.e., $J_{ij}\neq J_{ji}$. In this case no Hamiltonian can be defined.

The Hopfield model has helped to create the class of ANNs; systems where the fundamental states become stored memories. Each such memory defines a nontrivial phase space and possesses its own basin of attraction. In ANNs, an initial pattern that has a significant overlap with one stored memory—that is, belonging to its basin of attraction—evolves by means of an appropriate dynamics (stochastic or not) to a lower energy state, i.e., to the stored memory. This leads to the recognition of the pattern [1,2,3,4,20,21,22,23,24,25,26,27,28,29,30,31,32,33,34,35,36,37,38,39,40]. However, once a pattern is recognized, *the system stays in its resultant state forever*, even if the input pattern has acted only at the beginning of the process. Due to this aspect, it is easy to see that, of the items listed in the previous section, few are consistently fulfilled.

Three basic limitations that are common for ANNs are listed and discussed below. (i) They do not operate at the chaos–order border, as any complex system should [12,13,14,15], but rather on the ordered side; (ii) They present a maximum limit in their storage capacity; and (iii) They are inefficient when reacting to changes in the external environment.

The ordered side, where a multiplicity of low-energy states appears, is the main ingredient of an ANN; as a proposal for modeling one of the most complex systems in nature, restriction (i) means that the ANNs are very far from achieving an appropriate approach to the brain. The constraint (ii) comes from the fact that the memories correspond to ground states, each one having its own basin of attraction, so that the size of the basins reduces as the number of stored memories increases. This severely limits their number, even if these memories are not correlated. In general, the storage capacity of ANNs, with *N* neuronal units, may be expressed as p=αN, with α characterized by threshold values ($\alpha_{c}$); in most cases $\alpha_{c}$ is much smaller than one. In the standard Hopfield model, it is well-known that $\alpha_{c}≃0.14$ [22], so that the system becomes unable to recognize any further pattern for α>0.14. The limitation (iii) is related to the above-mentioned fact that, once a pattern is recognized, the system stays in its corresponding state; this feature prevents quick reactions to external changes. In order to consider such reactions in the standard Hopfield model, for $\alpha <\alpha_{c}$, one should change the initial state $\left\{\sigma_{i}\left(0\right)\right\}$, which may lead to a jump in phase space to a different basin of attraction. As will be shown later, for the model we define next, reactions that follow changes in the external environment occur naturally and smoothly.

Modifications to the Hopfield model, such as modifying coupling constants to be asymmetric (in which case there is no Hamiltonian) [41,42,43], correlating or coupling pairs of memories [44,45,46,47,48,49,50,51], and introducing dilution into the synapses [3,43,52,53,54,55,56,57,58,59,60,61,62], yield minor changes, but largely fail to satisfy the requirements for an appropriate description of the brain [steps (b)–(e) of the previous section], and do not overcome the drawbacks described in items (i)–(iii) above. Asymmetric coupling constants, correlations among memories, and dilution may lead to fixed points or cycles in the dynamics, which appear on the ordered side of the chaos–order dichotomy. These ingredients could also lead to a chaotic state [63,64]; in both situations (ordered or disordered states) one is not at the border of chaos and order, and thus the situation is inappropriate for emulating a complex system such as the brain.

## 3. Biologically Motivated Model: The Relevance of External Stimuli

Reacting quickly to changes in the neighboring environment is so important to life that many reactions are written into our DNA. Being the most primitive ones; they are commonly called instinctive reactions. The new-born mammal, which sucks anything that enters its mouth, illustrates an instinctive reaction, fundamental to the first weeks of any mammals’ life. Rapid and involuntary movements to escape from predators represent provide additional examples, among many other instinctive reactions in animals. Clearly, the brain was forged by evolution for the task of quickly analyzing any external stimulus and triggering a muscle reaction if necessary. With evolution, further types of reactions to external stimuli, non-instinctive reactions, were developed in the nervous systems of many animals. These animals can take some time to analyze the stimulus, recognize it, and then, react.

Prompt reactions to external stimuli should be ubiquitous for any brain activity; besides, of course, the important task of pattern recognition. During pattern recognition, in short, the system is able to store a certain number of patterns (memories), which come from previous experiences. If an external stimulus related to one of these patterns is presented, the system should be able to recognize it as one of the stored patterns.

The typical framework of using ANNs for pattern recognition generates a topography, in an appropriate mathematical space, where memories are at the bottom of the valleys and external stimuli are (initially) located on the mountain slopes, giving birth to the recognition process. An appropriate choice of dynamics should lead the external stimulus from the mountain slopes to the bottom of the valley, associating it with the corresponding memory.

In these types of ANN models, the influence of an external stimulus on a specific neuron belonging to the NN can be essentially split into two contributions; namely, a signal, connected with the external stimulus and correlated to a stored pattern (memory), as well as noise, produced by all stored memories that are not related to the external stimulus. This external stimulus yields the initial state of neuronal activity that, by evolving according to some internal process, leads the system to recognize the memory associated with the initial stimulus. As the number of stored patterns (memories) increases, the width of the noise distribution also increases, so that when the system attains a sufficiently large number of stored memories, this width becomes of the order of the signal; once this occurs, the NN is not able to recognize any further stored pattern. The ability of an ANN to recognize stored memories fails because the width of the noise distribution (roughly) cancels out the signal from the external initial stimulus.

In this paper, our main assumption is that the long-term evolution of living beings has allowed them to calibrate the external-stimulus influence, so that the noise produced by memories that are not correlated with the external stimulus is canceled as much as possible. Roughly, we can express the influence ($h_{i}$) due to other neurons (j≠i) on a particular neuron *i*, in the presence of an external stimulus correlated with some stored memory, in terms of the following contributions [65],

(4) $$h_{i}=\mathrm{signal}+\mathrm{noise}+\mathrm{external}\;\mathrm{stimulus}.$$

If the external stimulus succeeds in canceling the noise contribution, the signal should remain, allowing the recognition of the stimulus whenever it is associated to some stored pattern. The external stimulus does not merely correspond to an initial state, which is responsible for starting the process of recognition as happens in standard NNs [1,2,3,4], but it will rather be present during the whole process of recognition. In this framework, the initial state $\left\{\sigma_{i}\left(0\right)\right\}$ becomes irrelevant; the fundamental point concerns the process of tuning the external-stimulus intensity in order to cancel precisely (or approximately) the noise term. Operationally, in a given computational NN, the first two contributions on the right-hand-side of Equation (4) result from the model construction [e.g., from the definition of the intensity of synapses in Equations (1) and (2)], whereas the last contribution corresponds to an extra term acting on each neuron *i*, introduced in order to attenuate the noise contribution. From now on, we refer to this scheme as a stimulus-dependent neural network (SDNN).

In principle, this framework may be implemented in many NN models, but here it will be illustrated for the Hopfield model, due to its simplicity and potential for producing analytical results. In this case, the local field $h_{i}$ at time *t* becomes

(5) $$h_{i}\left(t\right)=\sum_{j\neq i}^{N}J_{ij}\;\sigma_{j}\left(t\right)+\kappa \;\eta_{i},$$

where, in the first term on the r.h.s., we have the usual contribution due to (N−1) neurons, whereas the second term corresponds to the external stimulus acting on neuron *i*. This later contribution should remain fixed during the whole time evolution, with $\eta_{i}=\pm 1$ and κ (κ≥0) depicting its intensity, to be considered in the recognition process. The main idea is to maintain the influence of the external pattern during the whole process, and not only as an initial state, as happens in typical ANNs.

We will consider two typical situations concerning this external stimulus: (i) It should present an overlap with one specific stored memory (e.g., memory ρ, 1≤ρ≤p). It should be orthogonal to all other memories and should lead to an external pattern being correlated with a specific memory; (ii) It should be orthogonal to all stored memories, possess no overlap with any memory, and correspond to an external pattern not correlated with any stored memory. Case (i) can be expressed by assuming that the set $\left\{\eta_{i}\right\}$ obeys the probability distribution

(6) $$P\left(\eta_{i}\right)=\gamma \delta \left(\eta_{i}-\xi_{i}^{\mu }\right)+\left(1-\gamma \right)\delta \left(\eta_{i}+\xi_{i}^{\mu }\right),$$

where 1/2<γ≤1 for μ=ρ and γ=1/2 for μ≠ρ. The particular case γ=1 means that the set of external-stimulus signs $\left\{\eta_{i}\right\}$ yields the same pattern as the memory ρ, covering common real situations where one should recognize an external pattern that coincides precisely with the stored one. On the other hand, case (ii) is characterized by

(7) $$P\left(\eta_{i}\right)=\frac{1}{2}\delta \left(\eta_{i}-\xi_{i}^{\mu }\right)+\frac{1}{2}\delta \left(\eta_{i}+\xi_{i}^{\mu }\right),\;\;\left(1\leq \mu \leq p\right).$$

Let us investigate the local field of Equation (5), by separating the contribution of memory ρ from those of other memories (μ≠ρ) in Equation (2); one obtains,

(8) $$h_{i}\left(t\right)=\frac{1}{N}\sum_{j\neq i}^{N}\xi_{i}^{\rho }\xi_{j}^{\rho }\sigma_{j}\left(t\right)+\frac{1}{N}\sum_{j\neq i}^{N}\sum_{\mu \neq \rho }^{p}\xi_{i}^{\mu }\xi_{j}^{\mu }\;\sigma_{j}\left(t\right)+\kappa \;\eta_{i}.$$

Comparing the equation above with Equation (4), one immediately identifies each of the contributions on its r.h.s.: the first one is the signal activated when the neuron configuration $\left\{\sigma_{i}\left(t\right)\right\}$ is roughly the same as the stored memory $\left\{\xi_{i}^{\rho }\right\}$; the second one represents the noise, induced by other memories distinct from ρ; and the third contribution corresponds to the external stimulus. In the case γ=1, if we choose $\sigma_{i}\left(t\right)=\eta_{i}=\xi_{i}^{\rho }$, then

(9) $$h_{i}\left(t\right)=\frac{N-1}{N}\;\xi_{i}^{\rho }+\frac{1}{N}\sum_{j\neq i}^{N}\sum_{\mu \neq \rho }^{p}\xi_{i}^{\mu }\xi_{j}^{\mu }\;\xi_{j}^{\rho }+\kappa \xi_{i}^{\rho },$$

which shows that one may calibrate κ appropriately, allowing the external stimulus to roughly cancel the noise term, in order to favor the signal contribution. This would allow the system to recognize the submitted external pattern. Therefore, κ must not be so small, since it will not be able to cancel the noise term, nor very large, in which case it may dominate the local field, forcing an alignment with the external stimulus, whether correlated with a particular memory or not. Finding an optimal value for κ is a fundamental point of this framework.

Next, we estimate, approximately, the optimal value of κ, by focusing on the noise contribution in Equation (9),

(10) $$h_{i}^{\mathrm{noise}}\left(t\right)=\frac{1}{N}\sum_{j\neq i}^{N}\sum_{\mu \neq \rho }^{p}\xi_{i}^{\mu }\xi_{j}^{\mu }\;\xi_{j}^{\rho }.$$

Considering that there are no correlations between patterns μ and ρ, and that patterns associated with different sites (j≠i) are independent, each of these contributions take the values ±1 with equal probability. For large enough values of *N* and *p* the average over the variables {ξ} is,

(11) $${\langle h_{i}^{\mathrm{noise}}\left(t\right)\rangle }_{\xi }≃0,$$

whereas its associated variance yields

(12) $${\langle {\left(h_{i}^{\mathrm{noise}}\left(t\right)\right)}^{2}\rangle }_{\xi }={\langle \frac{1}{N^{2}}\sum_{j\neq i}^{N}\sum_{\mu \neq \rho }^{p}\sum_{k\neq i}^{N}\sum_{\nu \neq \rho }^{p}\xi_{i}^{\mu }\xi_{j}^{\mu }\;\xi_{j}^{\rho }\xi_{i}^{\nu }\xi_{k}^{\nu }\xi_{k}^{\rho }\rangle }_{\xi }≃\frac{p}{N}=\alpha .$$

Hence, the width of the noise distribution is approximately ${\langle {\left(h_{i}^{\mathrm{noise}}\left(t\right)\right)}^{2}\rangle }_{\xi }^{1/2}∼\sqrt{\alpha }$, so that for independent patterns, a good choice for κ should roughly be $\sqrt{\alpha }$; the numerical simulations to be presented later on display good agreement with this choice. This procedure could indicate pattern recognition for values of *p* that are much higher than those restricted by the upper limit of the Hopfield model. However, in those models where analytical calculations for the optimal value of κ are not feasible, it should still be possible to estimate this value numerically; in fact, this is an easy task, as will be shown later on.

From Equation (8), with the reminder that the couplings $\left\{J_{ij}\right\}$ are symmetric in the Hopfield model, one can define a Hamiltonian at time *t*,

(13) $$\begin{array}{ccc} & & H\left(t\right)=-\sum_{i=1}^{N}h_{i}\left(t\right)\sigma_{i}\left(t\right)=-\frac{1}{N}\sum_{\left(i,j\right)}\xi_{i}^{\rho }\xi_{j}^{\rho }\sigma_{i}\left(t\right)\sigma_{j}\left(t\right)\\ & - & \frac{1}{N}\sum_{\left(i,j\right)}\sum_{\mu \neq \rho }^{p}\xi_{i}^{\mu }\xi_{j}^{\mu }\;\sigma_{i}\left(t\right)\sigma_{j}\left(t\right)-\kappa \;\sum_{i=1}^{N}\eta_{i}\;\sigma_{i}\left(t\right)\;, \end{array}$$

where $\sum_{\left(i,j\right)}$ denotes sums over all distinct pairs of neurons, corresponding to a fully connected NN.

In the simulations to be presented later, we essentially focused on two parameters, namely, the macroscopic superposition (or overlap) of a neuron state $\left\{\sigma_{i}\left(t\right)\right\}$ with a given pattern $\left\{\xi_{i}^{\mu }\right\}$ at time *t*,

(14) $$m_{\mu }\left(t\right)=\frac{1}{N}\sum_{i=1}^{N}\xi_{i}^{\mu }\sigma_{i}\left(t\right)\;\left(\mu =1,2,\ldots ,p\right),$$

as well as the overlap of this neuron state with the special case where the external-stimuli signs $\left\{\eta_{i}\right\}$ are orthogonal to all stored memories [i.e., case (ii) in Equation (7) with γ=1/2 for all memories], defined as

(15) $$m_{⊥}\left(t\right)=\frac{1}{N}\sum_{i=1}^{N}\eta_{i}\sigma_{i}\left(t\right).$$

The quantity above represents a macroscopic superposition with an external pattern, not stored and orthogonal to all stored patterns, for which $\eta_{i}=\pm 1$ with equal probability. Frequently, the set $\left\{m_{\mu }\left(t\right)\right\}$ is considered to contain the components of a *p*-dimensional vector, $\vec{m}\left(t\right)=\left(m_{1}\left(t\right),m_{2}\left(t\right),\ldots ,m_{\rho }\left(t\right),\ldots ,m_{p}\left(t\right)\right)$, and we will give a special emphasis to $m_{\rho }\left(t\right)$, as associated with a single condensed pattern and identified in the first term of the Hamiltonian in Equation (13). All other components $m_{\mu }\left(t\right)$(μ≠ρ) appear in the noise contribution of the Hamiltonian.

## 4. Analytic Calculations

In this section we perform analytical calculations, which essentially correspond to a mean-field approach, along the lines of Refs. [21,22]. We assume that the system defined by the Hamiltonian in Equation (13) attains, after a sufficiently long time, well-defined thermal equilibrium states (for finite temperatures, T>0), together with its zero-temperature limit (T→0). For a given realization of the quenched disorder $\left(\left\{\xi_{i}^{\mu }\right\},\left\{\eta_{i}\right\}\right)$, one may define a partition function $Z\equiv Z(\left\{\xi_{i}^{\mu }\right\},\left\{\eta_{i}\right\})$, so that the free energy per neuron becomes

(16) $$-\beta f=\lim_{N\to \infty }\frac{1}{N}\;{\langle \;\ln Z(\left\{\xi_{i}^{\mu }\right\},\left\{\eta_{i}\right\})\rangle }_{\eta ,\xi },$$

where β=1/T (we work in units $k_{B}=1$). Above, ${\langle \ldots \rangle }_{\eta ,\xi }$ indicates quenched averages on $\left\{\eta_{i}\right\}$ and $\left\{\xi_{i}^{\mu }\right\}$, which, according to Equations (3) and (6), should be carried out with the average over the $\left\{\eta_{i}\right\}$ taken before the one over $\left\{\xi_{i}^{\mu }\right\}$. Following standard procedure, we apply the replica method to calculate the free energy [1,2,3,4],

(17) $$-\beta f=\lim_{N\to \infty }\lim_{n\to 0}\frac{1}{Nn}\;\left({\langle Z^{n}\left(\left\{\xi_{i}^{\mu }\right\},\left\{\eta_{i}\right\}\right)\rangle }_{\eta ,\xi }-1\right),$$

where $Z^{n}\left(\left\{\xi_{i}^{\mu }\right\},\left\{\eta_{i}\right\}\right)$ corresponds to the partition function of *n* independent replicas, for a given realization of the disorder.

Then, one assumes that the total number of stored patterns is an extensive quantity, expressed as p=αN; in the standard Hopfield model, this holds up $\alpha_{c}≃0.14$ [22], so that pattern recognition does not work for α>0.14. At the above-mentioned equilibrium, one may perform the usual averages, so the quantity of Equation (14) leads to

(18) $$m_{\mu }={\langle \frac{1}{N}\sum_{i=1}^{N}\xi_{i}^{\mu }\langle \sigma_{i}\rangle \rangle }_{\eta ,\xi }\;\left(\mu =1,2,\ldots ,p\right),$$

whereas, for the case where the external stimulus is orthogonal to all stored memories, the superposition in Equation (15) yields

(19) $$m_{⊥}={\langle \frac{1}{N}\sum_{i=1}^{N}\eta_{i}\langle \sigma_{i}\rangle \rangle }_{\eta ,\xi },$$

with $\langle \sigma_{i}\rangle$ corresponding to local magnetizations.

Lengthy (but well-established) calculations for the free energy of Equation (17) are outlined in the Appendix A, where we have split the patterns $\left\{\xi_{i}^{\mu }\right\}$ into two sets; namely, *s* condensed patterns and (p−s) non-condensed ones. From now on, we will investigate the recognition of a single pattern μ=ρ (i.e., s=1), meaning that the external stimulus presents a superposition with memory ρ, corresponding to case (i) of Equation (6), for which one has the overlapping

(20) $$m_{\rho }={\langle \frac{1}{N}\sum_{i=1}^{N}\xi_{i}^{\rho }\langle \sigma_{i}\rangle \rangle }_{\eta ,\xi }.$$

Moreover, the free energy at the end of the Appendix A [cf. Equation (A7)] becomes

(21) $$\begin{array}{ccc} f & = & \frac{\alpha }{2}+\frac{1}{2}\;m_{\rho }^{2}+\frac{\alpha \beta }{2}r\left(1-q\right)+\frac{\alpha }{2\beta }\left[\ln\left(1-\beta +\beta q\right)-\frac{\beta q}{1-\beta +\beta q}\right]\\ & & \;\;\;\;-\frac{1}{\beta }\int Dz{\langle \ln\left[2\cosh\beta \left(z\sqrt{\alpha r}+m_{\rho }\xi^{\rho }+\kappa \eta \right)\right]\rangle }_{\eta ,\xi }, \end{array}$$

where

(22) $$\int Dz\;\ldots \equiv \int_{-\infty }^{\infty }\frac{dz}{\sqrt{2\pi }}\exp\left(-\frac{z^{2}}{2}\right)\ldots ,$$

and the averages ${\langle \ldots \rangle }_{\eta ,\xi }$ should be considered over the single condensed pattern $\xi^{\rho }$ only. Two further parameters appear in the above free energy, namely, the Edwards–Anderson one (which becomes relevant only for very low temperatures),

(23) $$q={\langle \frac{1}{N}\sum_{i=1}^{N}{\langle \sigma_{i}\rangle }^{2}\rangle }_{\eta ,\xi },$$

and a parameter that measures the noise produced by (p−1) non-condensed patterns,

(24) $$r=\frac{1}{\alpha N^{2}}\sum_{\mu \neq \rho }{\langle {\left(\sum_{i=1}^{N}\xi_{i}^{\mu }\langle \sigma_{i}\rangle \right)}^{2}\rangle }_{\eta ,\xi }.$$

In deriving the free energy in Equation (21), as well as the parameters in Equations (23) and (24), we have assumed the replica-symmetry ansatz: in full replica space, both parameters depend on two replica indices, representing matrix elements, as can be seen in the Appendix A. In this ansatz, one assumes that all off-diagonal matrix elements are equal, given by Equations (23) and (24) [1,2,3,4,18].

The equilibrium solution comes from the derivatives of the free-energy with respect to the parameters above, leading to the saddle-point equations,

(25) $$\begin{array}{ccc} m_{\rho } & = & \int Dz{\langle \xi^{\rho }\tanh\left[\beta \left(z\sqrt{\alpha r}+m_{\rho }\xi^{\rho }+\kappa \eta \right)\right]\rangle }_{\eta ,\xi }, \end{array}$$

(26) $$\begin{array}{ccc} q & = & \int Dz{\langle \tanh^{2}\left[\beta \left(z\sqrt{\alpha r}+m_{\rho }\xi^{\rho }+\kappa \eta \right)\right]\rangle }_{\eta ,\xi }, \end{array}$$

(27) $$\begin{array}{ccc} r & = & \frac{q}{{\left(1-\beta +\beta q\right)}^{2}}. \end{array}$$

For case (ii) in Equation (7), where the external stimulus is orthogonal to all memories, there is no condensed pattern, and the parameter $m_{⊥}$ of Equation (19) may be calculated from

(28) $$m_{⊥}=\frac{1}{\beta N}{\langle \frac{\partial }{\partial \kappa }\ln Z\left(\left\{\xi_{i}^{\mu }\right\},\left\{\eta_{i}\right\}\right)\rangle }_{\eta ,\xi }=\frac{1}{\beta N}\frac{\partial }{\partial \kappa }{\langle \;\ln Z(\left\{\xi_{i}^{\mu }\right\},\left\{\eta_{i}\right\})\rangle }_{\eta ,\xi }=-\frac{\partial f}{\partial \kappa },$$

where we have assumed, as usual, that the derivative comutes with the average operations, since these latter are essentially expressed by sums and integrals. It is important to stress that the averages above should be considered over the orthogonal patterns, for which $m_{\rho }=0$ and $\eta_{i}=\pm 1$ with equal probability. In this way,

(29) $$m_{⊥}=\int Dz{\langle \eta \tanh[\beta \left(z\sqrt{\alpha r}+\kappa \eta \right)]\rangle }_{\eta }.$$

### Limit T→0

Throughout this work, a special emphasis will be given to limit T→0 (or β→∞); in this case, q=1+O(T), so that the internal energy per neuron becomes

(30) $$u=\frac{1}{2}\;\alpha \left(1-r\right)-\frac{1}{2}\;m_{\rho }^{2}-\kappa m_{⊥}.$$

Additionally, Equations (25)–(29) lead to

(31) $$m_{\rho }=\gamma \;\mathrm{erf}\left(\frac{m_{\rho }+\kappa }{\sqrt{2\alpha r}}\right)+\left(1-\gamma \right)erf\left(\frac{m_{\rho }-\kappa }{\sqrt{2\alpha r}}\right),$$

(32) $$\begin{array}{c} r=\frac{1}{{\left(1-C\right)}^{2}};\;C=\sqrt{\frac{2}{\pi \alpha r}}\left\{\gamma \exp\left[-\frac{{\left(m_{\rho }+\kappa \right)}^{2}}{2\alpha r}\right]+\left(1-\gamma \right)\exp\left[-\frac{{\left(m_{\rho }-\kappa \right)}^{2}}{2\alpha r}\right]\right\}\;, \end{array}$$

with the case where the external stimulus is orthogonal to all stored memories yielding

(33) $$m_{⊥}=\mathrm{erf}\left(\frac{\kappa }{\sqrt{2\alpha r}}\right),$$

where erf(x) denotes the error function. Notice that in the particular limit γ=1, Equations (31) and (32) become

(34) $$m_{\rho }=\mathrm{erf}\left(\frac{m_{\rho }+\kappa }{\sqrt{2\alpha r}}\right),$$

and

(35) $$r=\frac{1}{{\left(1-C\right)}^{2}};\;C=\sqrt{\frac{2}{\pi \alpha r}}\exp\left[-\frac{{\left(m_{\rho }+\kappa \right)}^{2}}{2\alpha r}\right],$$

which, for κ=0, recover the zero-temperature equations of Ref. [22].

**Figure 1:**
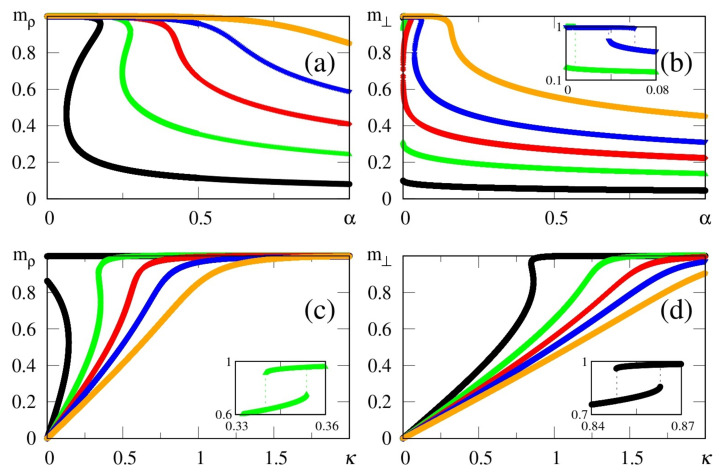
(Color online) Results from mean-field approximation in the zero-temperature limit, for the overlaps $m_{\rho }$ [Equation (34)] and $m_{⊥}$ [Equation (33)], are exhibited for the case γ=1: $m_{\rho }$ and $m_{⊥}$ are shown versus α (typical values of κ) in panels (**a**,**b**), and represented versus κ (typical values of α) in panels (**c**,**d**), respectively. In (**a**,**b**) one has κ=0.1,0.3,0.5,0.7, and 1.0 (from bottom to top), whereas in (**c**,**d**) one has α=0.1,0.3,0.5,0.7, and 1.0 (from top to bottom). For smaller values of κ and α, one notices multiple solutions for both $m_{\rho }$ and $m_{⊥}$, typical of first-order phase transitions. Insets illustrate some cases where two solutions coexist for certain intervals of these parameters: (**b**) κ=0.3 and 0.7; (**c**) α=0.3; (**d**) α=0.1.

The above zero-temperature equations may be solved numerically for given values of γ, α and κ; in Figure 1 we illustrate the particular case γ=1, with curves for the overlaps $m_{\rho }$ and $m_{⊥}$ resulting from the solution of Equations (33)–(35). The overlaps $m_{\rho }$ and $m_{⊥}$ versus α (typical values of κ), or versus κ (typical values of α), are presented in Figure 1a,b and Figure 1c,d, respectively. Here, we again emphasize that two different situations for the external stimulus are being considered, i.e., a fully correlated external stimulus (plots for $m_{\rho }$) and an external stimulus orthogonal to all memories (plots for $m_{⊥}$). One notices certain ranges of κ and α where multiple solutions for these quantities appear, characterizing first-order phase transitions. Some typical cases are illustrated in the corresponding insets, showing the coexistence of more than one solution; in these cases, the precise locations of the discontinuities may be computed through Maxwell constructions, where one equates the internal energy of Equation (30) for both solutions. For the purpose of pattern recognition, the quantities $m_{\rho }$ and $m_{⊥}$ are expected to vary smoothly, so that discontinuities, where one may have large variations on either one of these overlaps, for infinitesimal changes of given parameters, do not correspond to common situations in natural systems. Hence, for the rest of this work we will concentrate on solutions where the parameters $m_{\rho }$ and $m_{⊥}$, associated with two different types of external stimuli, vary smoothly on either one of the parameters κ or α; more particularly, we will focus on the role played by κ in the non-retrieval region of the standard Hopfield model, i.e., α>0.14, along which one has $m_{\rho }=m_{⊥}=0$ for κ=0. We call the attention to those plots for higher values of κ (or α) in Figure 1, focusing on the relevant regions for pattern recognition ($\kappa ≃\sqrt{\alpha }$), around which one notices significant values for the overlapping $m_{\rho }$ (typically, $m_{\rho }>0.8$).

## 5. Numerical Simulations: Recognition of Uncorrelated Patterns

We studied the SDNN, defined by the local field $h_{i}\left(t\right)$ of Equations (5)–(8), through zero-temperature numerical simulations, for $N=10^{4}$ neurons and p=αN stored memories, considering several choices for the parameters α and γ. At first, we set couplings and external stimuli according to Equations (2), (3) and (6); being quenched variables, these quantities were kept fixed along each time evolution. The initial neuron configuration was chosen at random, i.e., $\sigma_{i}\left(0\right)=\pm 1$ with equal probability; then, these dynamical variables were updated in a sequential way, through the zero-temperature dynamics,

(36) $$\sigma_{i}\left(t+1\right)=\mathrm{sign}\left[h_{i}\left(t\right)\right];\;\left(\forall i\right),$$

and each time unit corresponded to the operation above on a single neuron. Following this procedure, the nearest local minimum of energy is attained after a sufficiently long time $t^{*}$ ($t^{*}≃10^{2}N$, i.e., each neuron is visited $10^{2}$ times), with macroscopic quantities presenting slight fluctuations for $t>t^{*}$. In our analysis, we have emphasized the overlaps of Equations (19) and (20); at a given local minimum of energy, i.e., for $t>t^{*}$, these quantities can be calculated, and according to Equations (3) and (6), the average over $\left\{\eta_{i}\right\}$ should be taken before the one over $\left\{\xi_{i}^{\mu }\right\}$; for these averaging procedures, each simulation was repeated $10^{3}$ times.

Throughout this section, we restrict ourselves to the recognition of uncorrelated patterns; the satisfying performance of the present proposal in the case of correlated patterns will be shown later. In what follows, we will be particularly interested in the role played by κ in the non-retrieval region of the standard Hopfield model, where $m_{\rho }=m_{⊥}=0$ for κ=0. The intensity of the external stimulus κ>0 yields two distinct situations concerning the external pattern; namely, 1/2<γ≤1 for $m_{\rho }\left(\kappa \right)$ and γ=1/2 for $m_{⊥}\left(\kappa \right)$. According to this, sufficiently large values of κ lead to $m_{\rho }\to 2\gamma -1$ and $m_{⊥}\to 1$. However, these two particular limits (κ small or large) are not appropriate for pattern recognition; on the other hand, a well-calibrated value of κ, to be called hereafter $\kappa_{c}$, chosen in such a way to cancel (or diminish as much as possible) the effects of the noise contribution in Equation (8), as discussed in Section 3, represents the crucial point of this new framework. Values $\kappa \ll \kappa_{c}$ will not be sufficient to cancel the noise term, whereas for $\kappa \gg \kappa_{c}$, the external field will dominate the total local field and there will be no pattern recognition at all; in this later case, the local field will simply reproduce the external stimulus, whether associated with a memory or not. Here, we propose to monitor the absolute value of the difference between the overlaps of Equations (19) and (20), which correspond to two different types of external stimuli,

(37) $$\Delta m=|m_{\rho }-m_{⊥}|={\langle \frac{1}{N}\sum_{i=1}^{N}\left|\xi_{i}^{\rho }-\eta_{i}\right|\langle \sigma_{i}\rangle \rangle }_{\eta ,\xi },$$

in order to identify the optimal value $\kappa =\kappa_{c}$. One notices that only the orthogonal (i.e., non-stored) memories will contribute to Δm, so that the appropriate choice of $\kappa_{c}$ should correspond to its maximum value, max(Δm).

**Figure 2:**
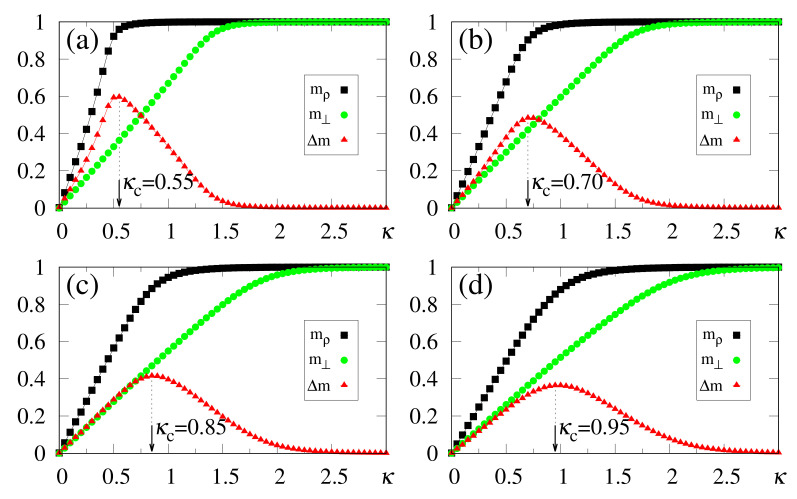
(Color online) Results from numerical simulations for the overlaps $m_{\rho }$ (black squares) [cf. Equation (20)], $m_{⊥}$ (green circles) [cf. Equation (19)], and the modulus of their difference, Δm (red triangles) [cf. Equation (37)], are plotted versus κ, for γ=1.0, and typical values of α: (**a**) α=0.4; (**b**) α=0.6; (**c**) α=0.8; (**d**) α=1.0. In each case, the maximum value of Δm yields the best choice for κ, denoted by $\kappa_{c}$. The two overlaps shown represent, respectively, macroscopic superpositions of a neuron state with a stored pattern ρ ($m_{\rho }$), and with non-stored patterns orthogonal to the stored one ($m_{⊥}$). The lines interpolating the symbols are guides for the eye.

Results from numerical simulations are shown in Figure 2, where the quantities defined in Equations (19), (20) and (37) are plotted versus κ, for γ=1.0 and typical values of α. In all cases, $\kappa_{c}$ is clearly identified by means of max(Δm), and since the storage capacity *p* grows with α, the variance of the noise contribution also increases. The analytical approximate result $\kappa_{c}≃\sqrt{\alpha }$ [cf. Equation (12)] shows good agreement with the numerical estimates of Figure 2. As another consequence of this increase, one finds a tendency towards diminishing the difference Δm, as well as the magnitude of the values max(Δm). In general, computing extremely accurate estimates for $\kappa_{c}$ is not a central aim in the present scheme, since slight variations around the values of max(Δm) provide equally good results. This is directly related to fluctuations in the noise contribution, shown in Section 3. It should be stressed that all values of α considered in Figure 2 are greater (or much greater) than the critical value $\alpha_{c}≃0.14$, above which the standard Hopfield NN does not recognize any pattern. Moreover, one can observe the limits $m_{\rho }=m_{⊥}=0$ (κ=0), as well as $m_{\rho }\to 2\gamma -1$ and $m_{⊥}\to 1$ ($\kappa \gg \kappa_{c}$).

**Figure 3:**
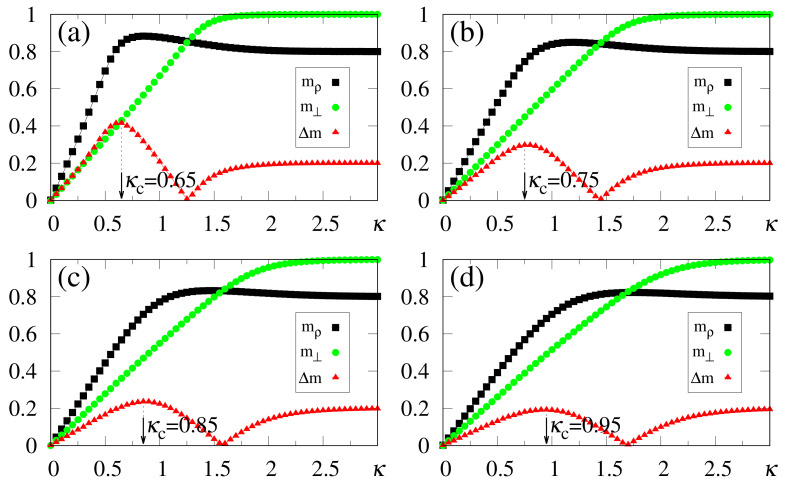
(Color online) Results from numerical simulations for the overlaps $m_{\rho }$ (black squares) [cf. Equation (20)], $m_{⊥}$ (green circles) [cf. Equation (19)], and the modulus of their difference, Δm (red triangles) [cf. Equation (37)], are plotted versus κ, for γ=0.9, and typical values of α: (**a**) α=0.4; (**b**) α=0.6; (**c**) α=0.8; (**d**) α=1.0. In each case, the maximum value of Δm yields the best choice for κ, denoted by $\kappa_{c}$. The two overlaps shown represent, respectively, macroscopic superpositions of a neuron state with a stored pattern ρ ($m_{\rho }$), and with non-stored patterns orthogonal to the stored one ($m_{⊥}$). The lines interpolating the symbols are guides for the eye.

An interesting effect appears in Figure 3, where we present results from numerical simulations for the quantities defined in Equations (19), (20) and (37) for γ=0.9, and the same values of α used in Figure 2. For $\kappa <\kappa_{c}$, all plots are qualitatively similar to those of Figure 2 and, in particular, very close estimates for $\kappa_{c}$ (or even equal in some cases) were obtained; one should notice that although the analytical result $\kappa_{c}≃\sqrt{\alpha }$ of Equation (12) was derived for the case γ=1, it holds for γ=0.9 as a good approximation. Nevertheless, the limits $m_{\rho }\to 2\gamma -1=0.8$ and $m_{⊥}\to 1$ are fulfilled for $\kappa \gg \kappa_{c}$, so that their plots cross ($m_{\rho }=m_{⊥}$) at some value of κ, yielding Δm=0. To the right of this crossing point one has that $m_{⊥}>m_{\rho }$, signaling that the external stimulus starts dominating the total local field, as one approaches a non-relevant limit for the SDNN. In this region, pattern recognition begins deteriorating, and for $\kappa \gg \kappa_{c}$ the local field will simply reproduce the external stimululs, whether associated with a memory or not. The optimal value $\kappa_{c}$, associated with the maximum difference between overlaps $m_{\rho }$ and $m_{⊥}$, represents the appropriate one, allowing the recognition of a stored pattern, for α>0.14; it cannot be too large, otherwise it prevents the external stimulus from dominating the other terms of the local field in Equation (8). This subtle balance is the heart of this approach.

One of the main virtues of the SDNN, illustrated in Figure 2 and Figure 3, concerns the range of α values, above the critical value of the standard Hopfield model, that still allow significant pattern recognition capabilities. Considering, as an illustrative example, the case α=1 [cf. Figure 2d and Figure 3d], one finds $\kappa_{c}≃0.95$ in both cases γ=1.0 and γ=0.9, and the macroscopic superposition with a given pattern ρ presents the values $m_{\rho }≃0.9$ [Figure 2d] and $m_{\rho }≃0.7$ [Figure 3d]. It should be noted that superpositions with memories μ≠ρ are still present, being of order $1/\sqrt{N}$. This enlargement in the pattern recognition capacity is directly related to the fact that the SDNN expresses no basins of attraction, although it creates a huge and single basin of attraction when $\kappa ≃\kappa_{c}$. However, by setting κ=0, this basin of attraction disappears and the memories do not occupy any volume in phase space, for any $\alpha >\alpha_{c}$. As will be shown later, within this framework recognition works well, even for values of α≫1.

**Figure 4:**
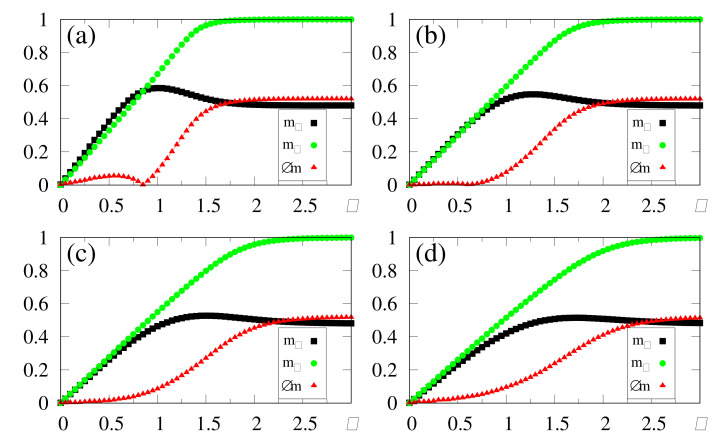
(Color online) Results from numerical simulations for the overlaps $m_{\rho }$ (black squares) [cf. Equation (20)], $m_{⊥}$ (green circles) [cf. Equation (19)], and the modulus of their difference, Δm (red triangles) [cf. Equation (37)], are plotted versus κ, for γ=0.74, and typical values of α: (**a**) α=0.4; (**b**) α=0.6; (**c**) α=0.8; (**d**) α=1.0. The two overlaps shown represent, respectively, macroscopic superpositions of a neuron state with a stored pattern ρ ($m_{\rho }$), and with non-stored patterns orthogonal to the stored one ($m_{⊥}$). The lines interpolating the symbols are guides for the eye.

As defined in Equation (6), the parameter γ measures the disturbance associated with recognition of a given stored pattern ρ. Therefore, as γ decreases from the value of one, we should find dissimilarities between the external stimulus and the stored pattern, so that pattern recognition fails for some γ<1. We illustrate this aspect in Figure 4, where we present a situation close to the limit of disturbance for the recognition of the stored pattern, γ≃0.74. One notices that there is not a clear optimal choice for κ in this case, so that one can not distinguish a stored pattern from a new pattern that is not stored and is orthogonal to other stored patterns. This feature of the model was investigated in Ref. [65], where this limit was verified, and it was shown that it is not possible to find an optimal choice of κ for γ≲0.74. Hence, in this approach, we can deform the stored pattern close to 25% and the SDNN will still recognize it, even for values of α greater than the critical limit of the Hopfield model. This peculiarity demonstrates an impressive performance compared with other NN models that are studied in the literature.

**Figure 5:**
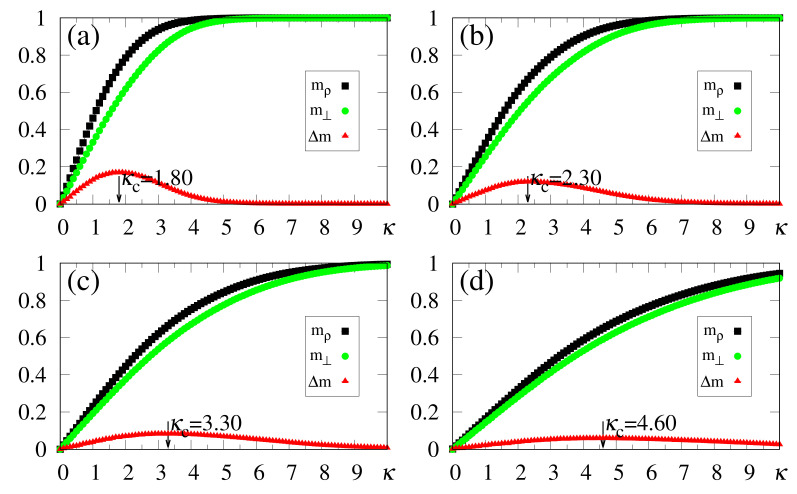
(Color online) Results from numerical simulations for the overlaps $m_{\rho }$ (black squares) [cf. Equation (20)], $m_{⊥}$ (green circles) [cf. Equation (19)], and the modulus of their difference, Δm (red triangles) [cf. Equation (37)], are plotted versus κ, for γ=1.0, and typical high values of α: (**a**) α=4; (**b**) α=8; (**c**) α=16; (**d**) α=32. In each case, the maximum value of Δm yields the best choice for κ, denoted by $\kappa_{c}$. The two overlaps shown represent, respectively, macroscopic superpositions of a neuron state with a stored pattern ρ ($m_{\rho }$), and with non-stored patterns orthogonal to the stored one ($m_{⊥}$). The lines interpolating the symbols are guides for the eye.

We have also carried out simulations that consider much higher values of α, with the external pattern corresponding to ρ, i.e., γ=1, as shown in Figure 5. One sees that there is no well-defined limit for the storage capacity, showing that the procedure described above holds for considerably large values of α. Particularly, as presented in Figure 5c, this technique was easily implemented for α=16; in this case we acquire $\kappa_{c}≃3.3$, together with $m_{\rho }≃0.7$, whereas the difference Δm remains quite significant (revealing a maximum value Δm≃0.1), and still allowing recognition. One should notice that the value of α used in Figure 5c is more than $10^{2}$ times larger than the threshold of the standard Hopfield model ($\alpha_{c}≃0.14$) [22]. Analyzing together the results of Figure 1 and Figure 5 (all of them for γ=1), two characteristics deserve discussion, as follows. (i) The analytical approximate result (derived for γ=1 and N→∞), $\kappa_{c}≃\sqrt{\alpha }$ [cf. Equation (12)], shows a good agreement with most numerical estimates (typically within 10% error), although the discrepancies increase for the larger α values. One sees that the analytical calculation yields overestimates for $\kappa_{c}$, with respect to the numerical results. These discrepancies are mostly due to the approximations leading to Equation (12), as well as to the finite sizes used in the simulations. (ii) Considering even larger values for α, a saturation effect occurs, in the sense that $m_{⊥}\to m_{\rho }$ from below, yielding Δm→0. This characteristic is illustrated in Figure 5d, where we consider α=32, which shows that the curves for $m_{\rho }$ and $m_{⊥}$ become very close, leading to a flat behavior in Δm. In this regime, clear identification of $\kappa_{c}$ becomes difficult, explaining the increase in the discrepancy between analytical and numerical results. We verified that such a saturation occurs, typically, for α≳40. Nevertheless, we can state that for relatively high values of α, as illustrated in Figure 5b,c, the SDNN is able to achieve pattern recognition with no difficulties.

**Figure 6:**
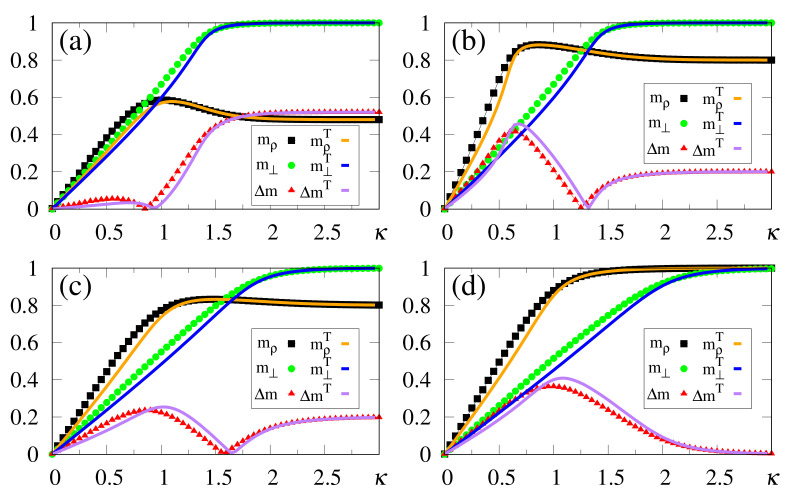
(Color online) Zero-temperature results from analytical calculations (full lines) are plotted versus κ and compared with those from numerical simulations (symbols) for typical values of α and γ: (**a**) α=0.4 and γ=0.74; (**b**) α=0.4 and γ=0.9; (**c**) α=0.8 and γ=0.9; (**d**) α=1.0 and γ=1.0. To distinguish from the numerical data, in each case the upper index T stands for theoretical results. The theoretical overlaps of Equation (33) (blue full line) and Equation (31) (brown full line) are compared with the numerical estimates for $m_{⊥}$ (green circles) [cf. Equation (19)] and $m_{\rho }$ (black squares) [cf. Equation (20)], respectively, whereas the theoretical modulus of the difference between these quantities (purple full line) is compared with the numerical Δm (red triangles) [cf. Equation (37)].

In Figure 6, we compare data from numerical simulations of $N=10^{4}$ neurons (symbols) with results from analytical calculations in the zero-temperature limit (full lines), considering typical choices for α and γ. The analytical results for $m_{⊥}$ follow from Equations (33) and (32), whereas those for $m_{\rho }$ were obtained from Equations (31) and (32); the corresponding numerical estimates were computed from Equations (19) and (20), respectively, with $\Delta m=|m_{\rho }-m_{⊥}|$ given by Equation (37). In all cases one notices a good agreement between the two approaches, with small discrepancies between them (at most of the order 0.05), which become more significant for small values of κ, as well as around the best choice $\kappa_{c}$, as expected, signaling the most relevant region for pattern recognition. However, the results from the two approaches essentially coincide when the external stimulus becomes very large, since in this limit the fluctuations in pattern recognition disappear. In Figure 6a, we present results for γ=0.74—i.e., very close to the limit of disturbance for the recognition of a stored pattern, as illustrated in Figure 5—so that there is not a well-defined value for $\kappa_{c}$. However, $\kappa_{c}$ may be obtained clearly from Figure 6b–d, and one notices in these cases that the analytical estimates (given by the maximum of Δm) are always overestimates with respect to the numerical ones [even in the cases γ=0.9 of Figure 6b,c]. This is in agreement with previous discussions referring to the approximations that led to Equation (12). The above-mentioned discrepancies are essentially due to finite-size effects, since analytical and numerical results are expected to coincide for N→∞, in which limit the mean-field approximation becomes exact for a fully connected network [1,2,3,4].

## 6. Time Evolution of the Macroscopic Superposition under Changes in the External Pattern

One well-known characteristic of living beings, fundamental to their survival, consists in their ability to react to changes in the environment. In this section, we show how the SDNN adjusts itself, being capable of replacing a previously recognized pattern with a new one, according to changes in the external stimulus. Let us consider a typical real situation where at a time $t_{0}$ we present a pattern associated with one stored memory, say memory ρ; then, at a later time $t_{1}$, we withdraw it and present a different pattern associated with memory ν. In order to illustrate the effectiveness of the SDNN in responding to these changes, we now allow the set of external-stimulus signs to change in time, i.e., $\left\{\eta_{i}\right\}\equiv \left\{\eta_{i}\left(t\right)\right\}$. Furthermore, we assume abrupt changes in these variables at two given times, $t=t_{0}$ and $t=t_{1}$, so that the probability distribution in Equation (6) is replaced by the time-dependent set

(38) $$P\left(\eta_{i}\left(t\right)\right)=\gamma_{1}\delta \left(\eta_{i}\left(t\right)-\xi_{i}^{\rho }\right)+\left(1-\gamma_{1}\right)\delta \left(\eta_{i}\left(t\right)+\xi_{i}^{\rho }\right);\;\left(t_{0}\leq t\leq t_{1}\right),$$

and

(39) $$P\left(\eta_{i}\left(t\right)\right)=\gamma_{2}\delta \left(\eta_{i}\left(t\right)-\xi_{i}^{\nu }\right)+\left(1-\gamma_{2}\right)\delta \left(\eta_{i}\left(t\right)+\xi_{i}^{\nu }\right);\;\left(t>t_{1}\right),$$

with $1/2\leq \gamma_{1},\gamma_{2}\leq 1$. The actions at times $t_{0}$ and $t_{1}$ correspond, respectively, to the presentation of pattern ρ, and to its replacement by the new pattern ν, giving rise to the question of whether the SDNN will respond adequately to these modifications.

**Figure 7:**
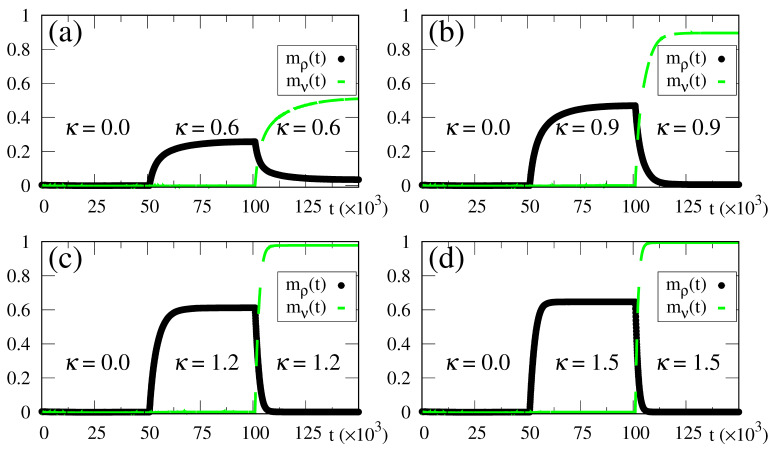
(Color online) Results from numerical simulations showing the time evolution of two different macroscopic superpositions of states of neurons with memories ρ and ν [$m_{\rho }\left(t\right)$ and $m_{\nu }\left(t\right)$, defined according to Equation (14)], for α=0.8. The external stimulus changes in time, following Equations (38) and (39), where the two relevant times are $t_{0}=5.0\times 10^{4}$ and $t_{1}=10^{5}$. Hence, for $t<t_{0}$ there is no stimulus, i.e., κ=0.0, and at $t=t_{0}$ a stimulus with an intensity κ appears with a superposition $\gamma_{1}=0.8$ with stored pattern $\left\{\xi^{\rho }\right\}$; then, at $t=t_{1}$ it changes abruptly, presenting a superposition $\gamma_{2}=1.0$ with stored pattern $\left\{\xi^{\nu }\right\}$. Four different intensities of the external stimulus are considered for $t>t_{0}$: (**a**) κ=0.6; (**b**) κ=0.9; (**c**) κ=1.2; (**d**) κ=1.5. The macroscopic superposition $m_{\nu }\left(t\right)$ (dashed green line) remains zero for $t<t_{1}$, whereas $m_{\rho }\left(t\right)$ (black circles) becomes nonzero for $t_{0}<t<t_{1}$.

In Figure 7, we illustrate how the SDNN reacts to the above-mentioned changes, by plotting the time-dependent macroscopic superpositions [cf. Equation (14)] $m_{\rho }\left(t\right)$ (black circles) and $m_{\nu }\left(t\right)$ (dashed green line) versus time, for α=0.8, while considering four different intensities of the external stimulus, in increasing order, as shown in Figure 7a–d. Up to $t_{0}=5.0\times 10^{4}$, no external pattern is presented (κ=0), and no memory is recognized, as expected. Then, a pattern with 80% ($\gamma_{1}=0.8$) superposition with the stored pattern $\left\{\xi^{\rho }\right\}$ is presented, leading to the onset of the macroscopic superposition $m_{\rho }\left(t\right)$ ($0\leq m_{\rho }\left(t\right)\leq 2\gamma_{1}-1$). Similarly, up to $t_{1}=10^{5}$, the macroscopic superposition $m_{\nu }\left(t\right)$ remains zero. It becomes nonzero only for $t>t_{1}$, due to the abrupt change in the stimulus at $t=t_{1}$, where pattern ρ is replaced by the new pattern, presenting a superposition $\gamma_{2}=1.0$ with stored pattern $\left\{\xi^{\nu }\right\}$. Accordingly, Figure 7 presents another important attribute of the SDNN—that is, its reactions to this change at $t=t_{1}$, resulting in a decrease in $m_{\rho }\left(t\right)$, together with a growth in $m_{\nu }\left(t\right)$ ($0\leq m_{\nu }\left(t\right)\leq 2\gamma_{2}-1$) for $t>t_{1}$.

Some interesting effects are shown in Figure 7, as discussed next. (i) The overlaps $m_{\rho }\left(t\right)$ and $m_{\nu }\left(t\right)$ approach a plateau whose height increases for larger values of κ, in agreement with the results presented in Figure 2, Figure 3, Figure 4, Figure 5 and Figure 6. (ii) The response time to the changes considered, either in the growth or reduction in the overlaps, is directly related to the intensity of the external stimulus κ: larger (smaller) values of κ lead to smaller (larger) response times. This type of behavior is also in agreement with reactions of living beings. In this way, one notices that for κ=0.6 the long-time limits for the overlaps have not been fully attained, as one can see by comparing the plot of $m_{\nu }\left(t\right)$ of Figure 7a with the long-time result of Figure 2c at κ=0.6 (both for α=0.8 and γ=1.0). One should note that the time interval for the action of the external stimulus in all panels of Figure 7 is $5.0\times 10^{4}$ (for both patterns ρ and ν), which is smaller than the time used in the simulations of Figure 2, Figure 3, Figure 4, Figure 5 and Figure 6, $t^{*}≃10^{2}N$ (i.e., $t^{*}≃10^{6}$), and thus sufficient for the SDNN to reach its nearest local minimum of energy. Due to this, for the smaller values of κ [cf., e.g., Figure 7a], both overlaps $m_{\rho }\left(t\right)$ and $m_{\nu }\left(t\right)$ have not yet reached their long-time limits. However, for larger values of κ, much smaller times are required for the overlaps $m_{\rho }\left(t\right)$ and $m_{\nu }\left(t\right)$ to approach their long-time limits; particularly, in Figure 7d, one notices that these overlaps have attained their maximum values; namely, $m_{\rho }\left(t\right)\to 2\gamma_{1}-1$ and $m_{\nu }\left(t\right)\to 2\gamma_{2}-1$. (iii) All panels of Figure 7 are illustrative, and exhibit the ability of the SDNN to react under changes in the environment. However, the most relevant interval for pattern recognition should be close to κ=0.9, as considered in Figure 7b, according to the values of $\kappa_{c}$ estimated in Figure 2c and Figure 3c.

One should note that reactions can also be studied in ANNs, as in the standard Hopfield model for $\alpha <\alpha_{c}$. In these cases, this may be achieved by changing the initial state $\left\{\sigma_{i}\left(0\right)\right\}$, which may lead to jumps in phase space among different basins of attraction. The results presented above show, clearly, that the SDNN modifies the recognized pattern, in a smooth way, according to changes in the external pattern, which is a common characteristic of living beings. In many real situations, living beings need to react to any new external pattern presented; this model behaves precisely in this way.

## 7. Recognition of Correlated Patterns

It is also interesting to investigate the performance of this framework when correlated patterns are stored. Let us suppose an external stimulus that presents a maximum overlap with one particular stored memory, let us say memory ρ, i.e., γ=1 in Equation (6). Then, we consider another pattern, θ, that presents a correlation *b* (0≤b≤1) with pattern ρ; this means that $\xi_{i}^{\rho }=\xi_{i}^{\theta }$ for a fraction *b* of indices *i*, leading to

(40) $$\frac{1}{N}\sum_{i=1}^{N}\xi_{i}^{\rho }\xi_{i}^{\theta }=b-\left(1-b\right)=2b-1\;\;\;\;\;\;\;\left(\rho \neq \theta \right).$$

Above, the limits b=0 and b=1 correspond to anti-correlated and fully correlated patterns, respectively, whereas the most interesting situations occur for 0<b<1.

**Figure 8:**
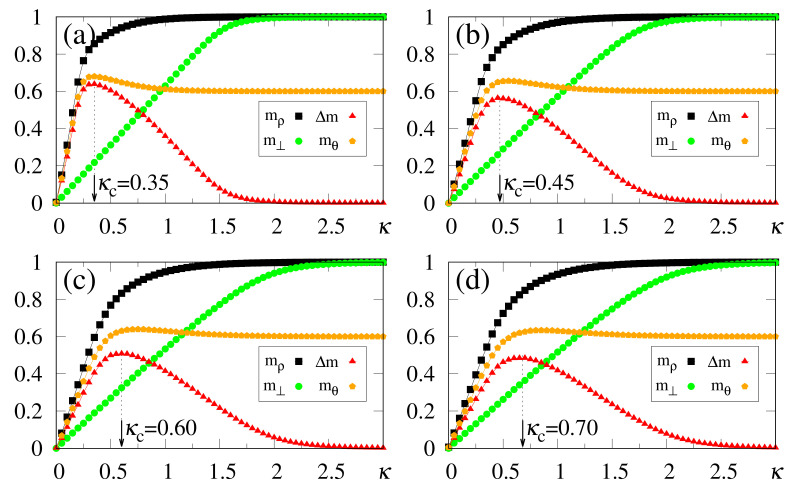
(Color online) Results from numerical simulations of two correlated patterns ($\left\{\xi_{i}^{\rho }\right\}$ and $\left\{\xi_{i}^{\theta }\right\}$), with between them present a correlation parameter b=0.8 [cf. Equation (40)], are plotted versus κ. The pattern ρ is fully correlated with the external stimulus (γ=1), and data for typical values of α are shown: (**a**) α=0.5; (**b**) α=0.7; (**c**) α=0.9; (**d**) α=1.0. The macroscopic superpositions with memories ρ and θ ($m_{\rho }$ and $m_{\theta }$) are represented by black squares and brown pentagons, respectively; the green circles are data for the macroscopic superposition $m_{⊥}$ [cf. Equation (19)], computed with a different external stimulus, orthogonal to all stored memories, whereas the red triangles stand for the modulus of the difference, $\Delta m=|m_{\rho }-m_{⊥}|$. In each case, the maximum value of Δm yields the best choice for κ, denoted by $\kappa_{c}$. The lines interpolating the symbols are guides for the eye.

In Figure 8, we present results from the simulations of two patterns, $\left\{\xi_{i}^{\rho }\right\}$ and $\left\{\xi_{i}^{\theta }\right\}$, with a correlation parameter b=0.8 between them; all other patterns are uncorrelated among themselves, as well as with these two. One may notice that the macroscopic superpositions, $m_{\rho }$ and $m_{\theta }$, attain the expected saturation limits for sufficiently large values of κ, i.e., $m_{\rho }\to 1$ (due to its maximum overlap with the external stimulus) and $m_{\theta }\to 0.6$ [following cf. Equation (40)]. Similarly to the previous situations investigated with uncorrelated patterns, the optimal value $\kappa_{c}$ increases for increasing values of α. Around their corresponding optimal values $\kappa_{c}$, the system recognizes both patterns, with $m_{\rho }≳0.8$ and $m_{\theta }$ already close to its saturation limit, $m_{\theta }≃0.6$, for all values of α considered, whereas all memories μ≠ρ,θ present overlaps that are essentially zero (in fact, of order $1/\sqrt{N}$). It is important to stress that even for α=1.0 one finds recognition of both stored patterns, indicating that pattern θ presents a significant overlap with the external stimulus, as a direct consequence of its correlation with the stored pattern ρ, demonstrating an appropriate feature of the SDNN model when dealing with correlated patterns. As another interesting result, one should call attention to the reduction of the estimates of $\kappa_{c}$, as a consequence of correlations; this aspect is revealed by comparing Figure 2d ($\kappa_{c}=0.95$) and Figure 8d ($\kappa_{c}=0.70$), both for γ=1 and α=1, for which one typically notices a 25% decrease on the value of $\kappa_{c}$.

**Figure 9:**
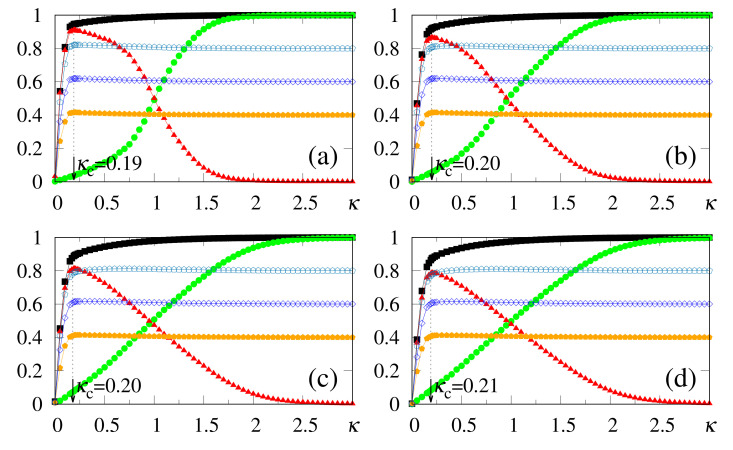
(Color online) Results from numerical simulations for three patterns, correlated with pattern $\left\{\xi_{i}^{\rho }\right\}$, are plotted versus κ. The pattern ρ is fully correlated with the external stimulus (γ=1) and data for typical values of α are shown: (**a**) α=0.5; (**b**) α=0.7; (**c**) α=0.9; (**d**) α=1.0. The macroscopic superposition with memory ρ, $m_{\rho }$, is represented by black squares, whereas those for the three correlated patterns, for values b=0.7,0.8, and 0.9, with respect to pattern $\left\{\xi_{i}^{\rho }\right\}$ [cf. Equation (40)], are depicted by brown pentagons, open blue diamonds, and open green pentagons, respectively (increasing values of *b* from bottom to top). The green circles are data for the macroscopic superposition $m_{⊥}$ [cf. Equation (19)], computed with a different external stimulus, orthogonal to all stored memories, whereas the red triangles stand for the modulus of the difference, $\Delta m=|m_{\rho }-m_{⊥}|$. In each case, the maximum value of Δm yields the best choice for κ, denoted by $\kappa_{c}$; one notices that $\kappa_{c}$ still increases with α, although much slower than in Figure 8. The lines interpolating the symbols are guides for the eye.

Results from the numerical simulations of several patterns—more precisely, $\left\{\xi_{i}^{\rho }\right\}$, and three other patterns correlated with $\left\{\xi_{i}^{\rho }\right\}$—are exhibited in Figure 9. The remaining (p−4) patterns are uncorrelated with these four and among themselves. Pattern $\left\{\xi_{i}^{\rho }\right\}$ presents a maximum overlap with the external stimulus (γ=1), whereas the three correlated ones follow Equation (40) with b=0.7,0.8, and 0.9. All macroscopic superpositions attain the expected saturation limits for sufficiently large values of κ, i.e., $m_{\rho }\to 1$, whereas the three correlated patterns approach their corresponding (2b−1) values, according to Equation (40). Notice that $\kappa_{c}≃0.2$ is sufficient to recover all four patterns, with $m_{\rho }≳0.9$ and the other three macroscopic superpositions already very close to their saturation limits, for all values of α considered; such significant values appear as direct consequences of the correlations with pattern $\left\{\xi_{i}^{\rho }\right\}$. A curious aspect of Figure 9 concerns the fact that the optimal value $\kappa_{c}$ increases much slower with α, when compared with previous cases investigated, for both uncorrelated patterns (see, e.g., Figure 2 and Figure 3) and two correlated patterns (cf. Figure 8). Besides small variations in $\kappa_{c}$, the magnitude of $\kappa_{c}$ is diminished by increasing the number of correlated patterns, as can be seen by comparing, e.g., Figure 2d ($\kappa_{c}=0.95$), Figure 8d ($\kappa_{c}=0.70$), and Figure 9d ($\kappa_{c}=0.21$), all of them for γ=1 and α=1. The present simulations suggest that, for a sufficiently large number of correlated patterns, $\kappa_{c}$ should converge to a small finite value $\kappa_{c}≳0$, for α fixed; by increasing α, a slow increase in the values of $\kappa_{c}$ should occur. This later result is in agreement with the recognition of similar patterns that is performed by living beings, where once one constituent of a given group is recognized, all similar members of the group are also recognized immediately, requiring little external stimulus for this task. The results of Figure 8 and Figure 9 illustrate additional important features of the SDNN model, presenting characteristics very similar to those of living beings in the recognition of correlated patterns.

**Figure 10:**
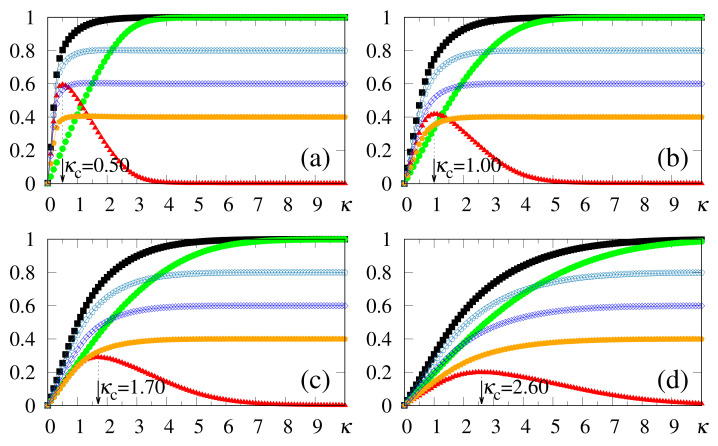
(Color online) Results from numerical simulations for three patterns, correlated with pattern $\left\{\xi_{i}^{\rho }\right\}$, are plotted versus κ. The pattern ρ is fully correlated with the external stimulus (γ=1) and data for typical values of α are shown: (**a**) α=2; (**b**) α=4; (**c**) α=8; (**d**) α=16. The macroscopic superposition with memory ρ, $m_{\rho }$, is represented by black squares, whereas those for the three correlated patterns, for values b=0.7,0.8, and 0.9, with respect to pattern $\left\{\xi_{i}^{\rho }\right\}$ [cf. Equation (40)], are depicted by brown pentagons, open blue diamonds, and open green pentagons, respectively (increasing values of *b* from bottom to top). The green circles are data for the macroscopic superposition $m_{⊥}$ [cf. Equation (19)], computed with a different external stimulus, orthogonal to all stored memories, whereas the red triangles stand for the modulus of the difference, $\Delta m=|m_{\rho }-m_{⊥}|$. In each case, the maximum value of Δm yields the best choice for κ, denoted by $\kappa_{c}$; one notices that $\kappa_{c}$ still increases with α. The lines interpolating the symbols are guides for the eye.

We have also tested how the SDNN behaves by considering several correlated patterns in the regime of large α values, as shown in Figure 10. Similarly to the experiments for Figure 8 and Figure 9, the estimates of $\kappa_{c}$ decrease for correlated patterns. This effect may be verified by comparing the results of Figure 10 (four correlated patterns) with those of Figure 5 (uncorrelated patterns), e.g., in the case α=4 [Figure 10b ($\kappa_{c}=1.0$) and Figure 5a ($\kappa_{c}=1.8$)], as well as α=16 [Figure 10d ($\kappa_{c}=2.6$) and Figure 5c ($\kappa_{c}=3.3$)], all for γ=1. This aspect is directly related to the gaps between the curves for $m_{\rho }$ and $m_{⊥}$, as can be seen from the corresponding above-mentioned plots. In these cases, one notices that the maximum value of Δm is typically doubled in Figure 10b,d, when contrasted to those of Figure 5a,c, respectively, making it easier to compute the values of $\kappa_{c}$. Such an increase in the maximum of Δm, together with the reduction in the values of $\kappa_{c}$, should yield an enlargement in the total number of memories, since the range of α values for pattern recognition is expanded; these results indicate that the saturation effect observed in Figure 5 should occur for even larger values of α, when one introduces correlations among patterns. Consequently, the introduction of such an important ingredient for real systems, i.e., correlations among patterns, is expected to improve, even further, the storage capacity of the SDNN.

## 8. Pattern Recognition in a Diluted Neural Network

In this section we show that the SDNN also performs well for diluted models; we illustrate this by analyzing its performance on the Hopfield model with non-symmetric synapse dilution. Let us then consider synaptic couplings in the modified form [52,53],

(41) $$J_{ij}=\frac{C_{ij}}{N(1-d)}\sum_{\mu =1}^{p}\xi_{i}^{\mu }\xi_{j}^{\mu },$$

where $\left\{C_{ij}\right\}$ are independent random variables following the probability distribution,

(42) $$P\left(C_{ij}\right)=\left(1-d\right)\;\delta \left(C_{ij}-1\right)+d\;\delta \left(C_{ij}\right)\;\left(C_{ij}\neq C_{ji}\right).$$

The variables $\left\{C_{ij}\right\}$ represent the asymmetric dilution of the couplings and d∈[0,1[ is a dilution parameter, defined as the fraction of the total number of connections that have been eliminated, i.e., a macroscopic dilution. Notice that the interactions $J_{ij}$ are not symmetric now, since for each neuron pair [$\sigma_{i}\left(t\right)$ and $\sigma_{j}\left(t\right)$], $C_{ij}$ and $C_{ji}$ are independent variables with $C_{ij}\neq C_{ji}$; consequently, no Hamiltonian can be defined. The two extremum values for the parameter *d* correspond to d=0 (undiluted limit), whereas its maximum value comes from Equation (41), leading to (1−d)∼1/N.

Next, to understand better the effects on $\kappa_{c}$ due to the dilution of synapses, let us estimate its value within the molecular-field approach; the procedure is similar to the one carried out in Section 3, through an analysis of the noise contribution. Therefore, the local field on neuron *i* is given by

(43) $$h_{i}\left(t\right)=\sum_{j\neq i}^{N}J_{ij}\sigma_{j}\left(t\right)+\kappa \eta_{i},$$

with $\left\{J_{ij}\right\}$ now given by Equation (41). Then, we assume that at a time *t* the neuron states $\left\{\sigma_{i}\left(t\right)\right\}$, as well as the external stimulus $\left\{\eta_{i}\right\}$, coincide with pattern $\left\{\xi_{i}^{\rho }\right\}$, leading to,

(44) $$\begin{array}{ccc} h_{i}\left(t\right) & = & \sum_{j\neq i}^{N}\sum_{\mu =1}^{p}\frac{C_{ij}}{N(1-d)}\xi_{i}^{\mu }\xi_{j}^{\mu }\xi_{j}^{\rho }+\kappa \xi_{i}^{\rho },\\ & = & \sum_{j\neq i}^{N}\frac{C_{ij}}{N(1-d)}\xi_{i}^{\rho }+\sum_{j\neq i}^{N}\sum_{\mu \neq \rho }^{p}\frac{C_{ij}}{N(1-d)}\xi_{i}^{\mu }\xi_{j}^{\mu }\xi_{j}^{\rho }+\kappa \xi_{i}^{\rho }. \end{array}$$

As before, the sum over memories μ was split into two contributions; namely, a signal produced by pattern μ=ρ, and a noise due to memories μ≠ρ,

(45) $$h_{i}^{\mathrm{noise}}\left(t\right)=\sum_{j\neq i}^{N}\sum_{\mu \neq \rho }^{p}\frac{C_{ij}}{N(1-d)}\xi_{i}^{\mu }\xi_{j}^{\mu }\xi_{j}^{\rho }.$$

Assuming that there are no correlations between patterns μ and ρ, and that patterns associated with different sites (j≠i) are independent, each memory contribution takes the values ±1 with equal probability. Noting that $\left\{C_{ij}\right\}$ are also independent variables, the noise random variables $\left\{h_{i}^{\mathrm{noise}}\left(t\right)\right\}$ do not yield a Gaussian for a finite *N*, but they should become Gaussian distributed for N→∞. Hence, considering large enough values of *N* and *p*, one acquires for the average over the variables {ξ},

(46) $${\langle h_{i}^{\mathrm{noise}}\left(t\right)\rangle }_{\xi }≃0,$$

whereas

(47) $${\left(h_{i}^{\mathrm{noise}}\left(t\right)\right)}^{2}=\frac{1}{{\left[N\left(1-d\right)\right]}^{2}}\sum_{j\neq i}^{N}\sum_{\mu \neq \rho }^{p}\sum_{k\neq i}^{N}\sum_{\nu \neq \rho }^{p}C_{ij}C_{ik}\xi_{i}^{\mu }\xi_{j}^{\mu }\;\xi_{j}^{\rho }\xi_{i}^{\nu }\xi_{k}^{\nu }\xi_{k}^{\rho }.$$

Since $\langle C_{ij}^{2}\rangle =\langle C_{ij}\rangle =1-d$, the associated variance with respect to the variables {ξ} becomes

(48) $${\langle {\left(h_{i}^{\mathrm{noise}}\left(t\right)\right)}^{2}\rangle }_{\xi }≃\frac{\left(N-1\right)\left(p-1\right)}{N^{2}\left(1-d\right)}≃\frac{p}{N(1-d)}=\frac{\alpha }{1-d}.$$

Similarly to the discussion carried out in Section 3, one should choose $\kappa_{c}$ equal to the width of the noise distribution,

(49) $$\kappa_{c}={\langle {\left(h_{i}^{\mathrm{noise}}\left(t\right)\right)}^{2}\rangle }_{\xi }^{1/2}\;≃\sqrt{\frac{\alpha }{1-d}},$$

in order to cancel (approximately) the noise contribution. According to this, a dilution parameter 0<d<1 yields an increase in the analytical approximate value for $\kappa_{c}$, as compared to the undiluted case d=0; this effect is in agreement with numerical simulations, as will be shown below. The limit $d\to 1-N^{-1}$, i.e., a fully diluted system, is not relevant for living beings and will not be investigated here; in this case one should have $\kappa_{c}\to \infty$.

**Figure 11:**
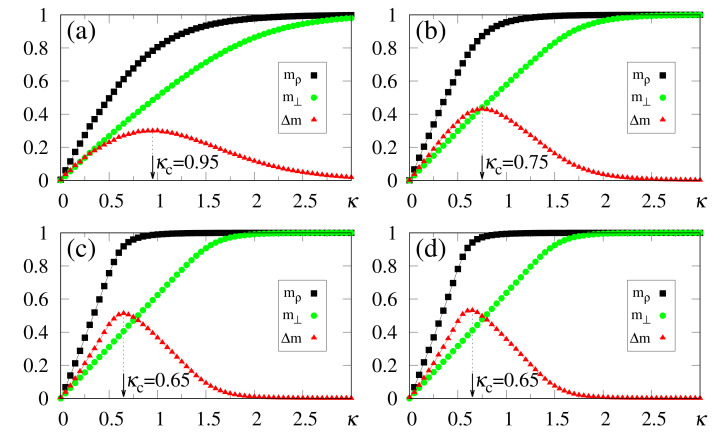
(Color online) Results from numerical simulations for the overlaps $m_{\rho }$ (black squares) [cf. Equation (20)], $m_{⊥}$ (green circles) [cf. Equation (19)], and the modulus of their difference, Δm (red triangles) [cf. Equation (37)], are plotted versus κ, for γ=1.0, α=0.5, and typical values of the dilution parameter *d*: (**a**) d=0.7; (**b**) d=0.4; (**c**) d=0.1; (**d**) d=0.0. In each case, the maximum value of Δm yields the best choice for κ, denoted by $\kappa_{c}$. The two overlaps shown represent, respectively, macroscopic superpositions of a neuron state with a stored pattern ρ ($m_{\rho }$), and with non-stored patterns orthogonal to the stored one ($m_{⊥}$). The lines interpolating the symbols are guides for the eye.

**Figure 12:**
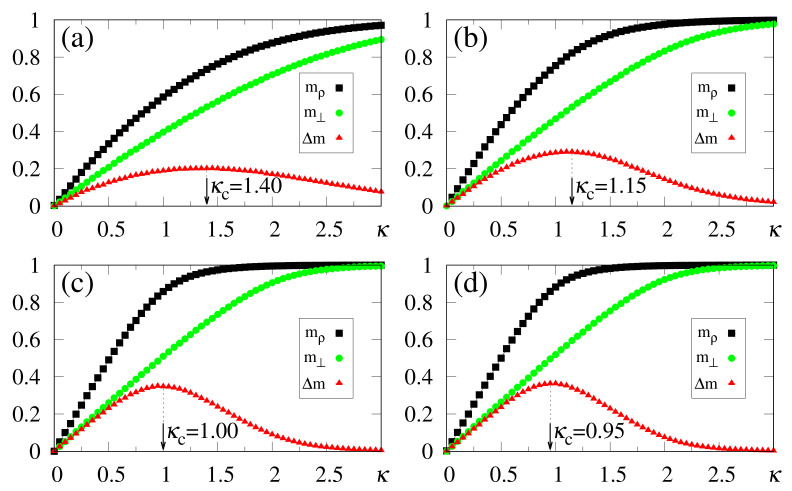
(Color online) Results from numerical simulations for the overlaps $m_{\rho }$ (black squares) [cf. Equation (20)], $m_{⊥}$ (green circles) [cf. Equation (19)], and the modulus of their difference, Δm (red triangles) [cf. Equation (37)], are plotted versus κ, for γ=1.0, α=1.0, and typical values of the dilution parameter *d*: (**a**) d=0.7; (**b**) d=0.4; (c) d=0.1; (**d**) d=0.0. In each case, the maximum value of Δm yields the best choice for κ, denoted by $\kappa_{c}$. The two overlaps shown represent, respectively, macroscopic superpositions of a neuron state with a stored pattern ρ ($m_{\rho }$), and with non-stored patterns orthogonal to the stored one ($m_{⊥}$). The lines interpolating the symbols are guides for the eye.

In what follows, for simplicity, we illustrate the effects of synapse dilution for uncorrelated patterns, considering γ=1.0 in Equation (6), i.e., an external pattern coinciding precisely with the stored one. Results from numerical simulations are shown in Figure 11 and Figure 12, with the quantities defined in Equations (19), (20) and (37) being plotted versus κ, for decreasing values of the dilution parameter *d*, and two typical choices of α, namely, α=0.5 [Figure 11] and α=1.0 [Figure 12]. One notices that the limits $m_{\rho }=m_{⊥}=0$ (κ=0) still hold; furthermore, for sufficiently high values of κ, the saturation limits $m_{\rho }\to 2\gamma -1$ and $m_{⊥}\to 1$ should be approached, even for high dilutions. One important aspect in these figures concerns the fact that $\kappa_{c}$ is clearly identified by means of max(Δm), even for the largest value of *d* considered [d=0.7 in Figure 11a and Figure 12a]. Moreover, for fixed choices of the dilution parameter *d*, $\kappa_{c}$ increases with α, as already observed for the undiluted case, whereas for fixed α, one notices that $\kappa_{c}$ decreases by decreasing *d*; these results are in agreement with the theoretical estimate of Equation (49). However, the discrepancies between the numerical and analytical estimates for $\kappa_{c}$ are larger for 0<d<1, when compared to those for the undiluted limit (d=0); whereas the relative discrepancies in the undiluted limit are typically of the order 10%, for larger dilutions, e.g., d=0.7, one may have relative discrepancies slightly larger than 20%. Similarly to the large α limit of the undiluted case [see, e.g., Figure 5] the curves for Δm become flatter for high dilutions, leading to such larger discrepancies. Furthermore, as before, the analytical result of Equation (49) represents overestimates with respect to the numerical ones, for all cases shown in Figure 11 and Figure 12. Increasing values of $\kappa_{c}$ with dilution is qualitatively expected, since synapse dilution diminishes the possible paths for transmitting given information, and so, larger intensities of the external field should be necessary in the pattern-recognition process.

## 9. General Discussion and Potential Applications

In contrast to standard ANNs, where memories are associated with minima in a metaphorical energy landscape, in the present SDNN model a given minimum appears only after a stimulus correlated with a stored pattern is presented. Such a stimulus is inspired by the form of a random magnetic field, commonly used on models of magnetism, acting independently on each neuron; it is expressed as $\kappa \eta_{i}$ [$\eta_{i}=\pm 1$; κ≥0] and kept active during the entirety of the recognition process.

The crucial part of the scheme is to calibrate the intensity of the external stimulus κ in order to cancel out, as much as possible, the noise contribution due to other patterns, which are not correlated with the external pattern presented. We developed a technique for calculating this optimal value (referred to as $\kappa_{c}$), which consists in estimating the maximum gap between: i) the macroscopic superpositions of a neuron state with a stored pattern ρ ($m_{\rho }$), and ii) those with non-stored patterns orthogonal to the stored one ($m_{⊥}$). More specifically, the maximum of $\Delta m=|m_{\rho }-m_{⊥}|$ yields $\kappa_{c}$, and it was shown that, around this value, the overlap $m_{\rho }$ attained significant values, making the model appropriate for pattern recognition.

We showed that the SDNN considerably increases the capability of the NN to recognize previously stored patterns. The proposal was illustrated through the inclusion of the additional contribution $\kappa \eta_{i}$ to the standard Hopfield model, for which the number of stored patterns is expressed as p=αN (*N* representing the total number of neurons), and is known to be unable to recognizing any further patterns for α≳0.14. Taking into account that analytical calculations may be performed for the Hopfield model, we compared analytical and numerical results, showing good agreement between the two approaches. In contrast to the standard Hopfield case, we found no threshold value for α. Rather, a saturation effect for sufficiently large α was observed, in the sense that the two overlaps, $m_{\rho }$ and $m_{⊥}$, become very close, resulting in Δm→0. In spite of this, we verified a significant increase in the recognition capacity of the neural network, so that for this specific application, considering no correlations between stored patterns, the range of possible values of α is enlarged, typically, by a factor $10^{2}$.

The impressive performance of the present proposal was also demonstrated in situations designed to mimic the common daily tasks of living beings, as described next. (i) Its ability to react promptly to changes in the external environment, reproducing a fundamental characteristic of living beings which need to react quickly to newly presented external patterns. (ii) Its recognition of correlated patterns, showing that correlations lead to a decrease in the optimal values of $\kappa_{c}$, together with an increase in the maxima of Δm; as a direct consequence, the numerical simulations indicate that the range of α values become enlarged, due to these correlations. These results are in agreement with the recognition of groups of patterns performed by living beings; once one constituent of a given group is recognized, all similar members of this group are identified immediately, and this process requires only a small external stimulus. (iii) Its correct functioning for both asymmetric and diluted synapses, showing that when dilution is included, higher values of the external stimulus are necessary.

It is important to stress that the features of the present SDNN can be implemented both computationally (i.e., in software), as well as on devices. Computationally, there are many modern algorithms that use various gates (such as AND or XOR), and which implement Hebb-like rules (see, e.g., Refs. [66,67,68,69]). In line with these algorithms, the synapse values $\left\{J_{ij}\right\}$ may be updated whenever new memories are added to the network. In the present proposal, synapses are responsible for the first two terms in the right-hand-side of Equations (8) and (9), so that by changing their values, these two contributions are modified. To introduce the external-pattern contribution [third term of Equations (8) and (9)], one should add the signal of the external stimulus $\kappa \eta_{i}$ to each neuron *i*. Hence, neurons will be updated in a sequential way, through the zero-temperature dynamics, $\sigma_{i}\left(t+1\right)=\mathrm{sign}\left[h_{i}\left(t\right)\right]$. In a given device, Hebb’s rule can be implemented in several ways by means of neuromorphic engineering, which is a recently developed area of research (see, e.g., Refs. [70,71]).

## 10. Conclusions

Based on the common behavior of living beings, we proposed a new neural-network framework, in which an external stimulus exerts a strong influence on the pattern-recognition process. This external stimulus, introduced in the form of a random field, remains active during the whole recognition process, considerably increasing the capability of the neural network to recognize previously stored patterns. In contrast to more-common attractor neural networks in the absence of an external field, memories are not attractors inside basins of attraction, and basins can be generated for external stimuli that present significant macroscopic superpositions with stored memories.

Finally, it is important to mention that this procedure may be implemented upon a large diversity of neural-network models, utilizing both analytical and numerical investigations. Moreover, its potential application in other processes, distinct from pattern recognition, is very appealing. The present proposal should help to shorten the wide gap between the performance of many of these NN models and the common characteristics of real living beings, improving their performance and, in particular, leading to a considerable increase in their recognition and reaction capabilities. Furthermore, the application of the presented theoretical concepts, both computationally (i.e., in software), as well as on physical devices, is a very promising prospect, and we hope that this task is taken up in the near future.

## Data Availability

Data is contained within the article.

## Appendix A. Mean-Field Equations

In this Appendix A, we detail some of the analytic calculations of Section 4; in particular, those leading to the free energy of Equation (21). We follow the lines of Refs. [21,22], where one assumes that the total number of memories *p* increases linearly with *N*, by introducing α=p/N as a finite number. In order to calculate the average over the quenched disorder, one applies the replica method, as defined in Equation (17), where $Z^{n}\left(\left\{\xi_{i}^{\mu }\right\},\left\{\eta_{i}\right\}\right)$ corresponds to the partition function of *n* independent replicas for a given realization of the disorder [1,2,3,4]. One has that

(A1) $${\langle Z^{n}\rangle }_{\eta ,\;\xi }={\langle \underset{{}_{\left\{\sigma^{a}\right\}}}{\mathrm{Tr}}\left[\prod_{a=1}^{n}\exp\beta \left(\frac{1}{N}\sum_{\left(i,j\right)}\sum_{\mu =1}^{p}\xi_{i}^{\mu }\xi_{j}^{\mu }\sigma_{i}^{a}\sigma_{j}^{a}+\sum_{i}\kappa \eta_{i}\sigma_{i}^{a}\right)\right]\rangle }_{\eta ,\;\xi },$$

where $\sum_{\left(i,j\right)}$ denotes a sum over all distinct pairs of neurons, corresponding to a fully connected neural network; in this case, the mean-field approach becomes exact in the thermodynamic limit. Moreover, *a* tags the *n* replicas (a=1,2,…,n) and ${\mathrm{Tr}}_{\left\{\sigma^{a}\right\}}$ stands for a trace over the neuron states, for each of the *n* replicas.

Now, using the Gaussian integral

(A2) $$\exp\left(\lambda a^{2}\right)=\frac{1}{\sqrt{2\pi }}\int_{-\infty }^{\infty }\;dx\exp\left[-x^{2}/2+\sqrt{2\lambda }ax\right],$$

one transforms the neuron-pair contributions (i,j) into single-neuron ones, by introducing integration variables $m_{\mu }^{a}$. Splitting the *p* patterns into two sets, namely, condensed $\left(\mu =1,2,\ldots ,s\right)$, and non-condensed $\left(\mu =s+1,s+2,\ldots ,p\right)$ patterns, one may calculate the averages over the non-condensed patterns $\left(\mu >s\right)$,

(A3) $$\begin{array}{ccc} {\langle Z^{n}\rangle }_{\eta ,\;\xi } & = & e^{-\beta pn/2}\langle \underset{{}_{\left\{\sigma^{a}\right\}}}{\mathrm{Tr}}\int \prod_{a,\;\mu }\frac{dm_{\mu }^{a}}{\sqrt{2\pi }}\exp\left(-\sum_{\begin{array}{c} \mu ,\;a\\ \left(\mu >s\right) \end{array}}\frac{{\left(m_{\mu }^{a}\right)}^{2}}{2}+\sum_{\begin{array}{c} i,\;\mu \\ \left(\mu >s\right) \end{array}}\ln\left(\cosh\sqrt{\frac{\beta }{N}}\sum_{a}m_{\mu }^{a}\sigma_{i}^{a}\right)\right)\\ & & {\left.\times \exp\left(-\sum_{\begin{array}{c} \mu ,\;a\\ \left(\mu \leq s\right) \end{array}}\frac{{\left(m_{\mu }^{a}\right)}^{2}}{2}+\sum_{\begin{array}{c} \mu ,\;a\\ \left(\mu \leq s\right) \end{array}}\sqrt{\frac{\beta }{N}}m_{\mu }^{a}\sum_{i}\xi_{i}^{\mu }\sigma_{i}^{a}+\beta \kappa \sum_{i,\;a}\eta_{i}\sigma_{i}^{a}\right)\right)\rangle }_{\eta ,\;\xi }. \end{array}$$

Assuming that for non-condensed patters $m_{\mu }^{a}=O\left(1\right)$, one uses the approximation $\ln\left[\cosh x\right]\approx \frac{1}{2}x^{2}$ and integrates over $m_{\mu }^{a}$ for μ>s, leading to

(A4) $$\begin{array}{ccc} & & \langle Z^{n}\rangle =e^{-\beta pn/2}\;\underset{{}_{\left\{\sigma^{a}\right\}}}{\mathrm{Tr}}\exp\left\{-\frac{p}{2}\;\mathrm{Tr}\ln\left[\left(1-\beta \right)I-\beta q\right]\right\}\\ & & \times {\langle \int \prod_{\mu ,\;a}{\left(\frac{N\beta }{2\pi }\right)}^{1/2}dm_{\mu }^{a}\exp\left[\beta N\left(-\frac{1}{2}\sum_{\mu ,\;a}{\left(m_{\mu }^{a}\right)}^{2}+\frac{1}{N}\sum_{i,\;\mu ,\;a}m_{\mu }^{a}\xi_{i}^{\mu }\sigma_{i}^{a}+\frac{\kappa }{N}\sum_{i,\;a}\eta_{i}\sigma_{i}^{a}\right)\right]\rangle }_{\eta ,\;\xi } \end{array}$$

where the change $m_{\mu }^{a}\to {\left(N\beta \right)}^{1/2}m_{\mu }^{a}$ was used. The matrix q is defined by the elements $q_{ab}=\frac{1}{N}\sum_{i}\sigma_{i}^{a}\sigma_{i}^{b}-\delta_{ab}$, and from now on, the index μ applies to condensed states (μ=1,2,…,s). The integrals over the set $\left\{m_{\mu }^{a}\right\}$ are evaluated by means of the steepest-descent method; introducing $r_{ab}$ as Lagrange multipliers for the non-diagonal elements $\left\{q_{ab}\right\}$, one has

(A5) $$f=\frac{\alpha }{2}+\lim_{n\to 0}\frac{1}{n}\;g_{n}\left(\left\{m_{\mu }^{a}\right\},\left\{q_{ab}\right\},\left\{r_{ab}\right\}\right),$$

with the functional (in full replica space) $g_{n}\left(\left\{m_{\mu }^{a}\right\},\left\{q_{ab}\right\},\left\{r_{ab}\right\}\right)$ given by

(A6) $$\begin{array}{ccc} & & g_{n}\left(\left\{m_{\mu }^{a}\right\},\left\{q_{ab}\right\},\left\{r_{ab}\right\}\right)=\frac{1}{2}\sum_{\mu ,\;a}{\left(m_{\mu }^{a}\right)}^{2}+\frac{\alpha }{2\beta }\mathrm{Tr}\ln\left[\left(1-\beta \right)I-\beta q\right]+\frac{\alpha \beta }{2}\sum_{\begin{array}{c} a,\;b\\ \left(a\neq b\right) \end{array}}r_{ab}q_{ab}\\ & & -\frac{1}{\beta }{\langle \ln\left[\underset{{}_{\left\{\sigma^{a}\right\}}}{Tr}\exp\left(\frac{\alpha \beta^{2}}{2}\sum_{a\neq b}r_{ab}\sigma^{a}\sigma^{b}+\beta \sum_{\mu ,\;a}m_{\mu }^{a}\xi^{\mu }\sigma^{a}+\beta \kappa \sum_{a}\eta \sigma^{a}\right)\right]\rangle }_{\eta ,\;\xi }. \end{array}$$

Now, one assumes the replica-symmetry ansatz [4], by imposing all off-diagonal elements of the matrix q (as well as their corresponding Lagrange multipliers) to be equal, i.e., $q_{ab}=q$ and $r_{ab}=r$ (a≠b). Considering the limit n→0, the free energy in Equation (A5) may be written as

(A7) $$\begin{array}{ccc} f & = & \frac{\alpha }{2}+\frac{1}{2}\sum_{\mu =1}^{s}{\left(m_{\mu }\right)}^{2}+\frac{\alpha \beta }{2}r\left(1-q\right)+\frac{\alpha }{2\beta }\left[\ln\left(1-\beta +\beta q\right)-\frac{\beta q}{1-\beta +\beta q}\right]\\ & & \;\;\;\;-\frac{1}{\beta }\int Dz{\langle \ln\left[2\cosh\beta \left(z\sqrt{\alpha r}+\sum_{\mu =1}^{s}m_{\mu }\xi^{\mu }+\kappa \eta \right)\right]\rangle }_{\eta ,\xi }, \end{array}$$

where

(A8) $$\int Dz\;...\equiv \int_{-\infty }^{\infty }\frac{dz}{\sqrt{2\pi }}\exp\left(-\frac{z^{2}}{2}\right)\ldots .$$
